# Eighty-four per cent of all Amazonian arboreal plant individuals are useful to humans

**DOI:** 10.1371/journal.pone.0257875

**Published:** 2021-10-01

**Authors:** Sara D. Coelho, Carolina Levis, Fabrício B. Baccaro, Fernando O. G. Figueiredo, André Pinassi Antunes, Hans ter Steege, Marielos Peña-Claros, Charles R. Clement, Juliana Schietti

**Affiliations:** 1 Programa de Pós-graduação em Ecologia, Instituto Nacional de Pesquisas da Amazônia, Manaus, Amazonas, Brazil; 2 Forest Ecology and Forest Management Group, Wageningen University & Research, Wageningen, The Netherlands; 3 Programa de Pós-Graduação em Ecologia, Universidade Federal de Santa Catarina, Florianópolis, Santa Catarina, Brazil; 4 Departamento de Biologia, Universidade Federal do Amazonas, Manaus, Amazonas, Brazil; 5 Coordenação de Biodiversidade, Instituto Nacional de Pesquisas da Amazônia, Manaus, Amazonas, Brazil; 6 RedeFauna - Rede de Pesquisa em Diversidade, Conservação e Uso da Fauna da Amazônia, Manaus, Amazonas, Brazil; 7 Coordenação de Dinâmica Ambiental, Instituto Nacional de Pesquisas da Amazônia, Manaus, Amazonas, Brazil; 8 Naturalis Biodiversity Center, Leiden, The Netherlands; 9 Systems Ecology, Vrije Universiteit Amsterdam, Amsterdam, The Netherlands; 10 Coordenação de Tecnologia e Inovação, Instituto Nacional de Pesquisas da Amazônia, Manaus, Amazonas, Brazil; Universidad Mayor de San Andrés, PLURINATIONAL STATE OF BOLIVIA

## Abstract

Plants have been used in Amazonian forests for millennia and some of these plants are disproportionally abundant (hyperdominant). At local scales, people generally use the most abundant plants, which may be abundant as the result of management of indigenous peoples and local communities. However, it is unknown whether plant use is also associated with abundance at larger scales. We used the population sizes of 4,454 arboreal species (trees and palms) estimated from 1946 forest plots and compiled information about uses from 29 Amazonian ethnobotany books and articles published between 1926 and 2013 to investigate the relationship between species usefulness and their population sizes, and how this relationship is influenced by the degree of domestication of arboreal species across Amazonia. We found that half of the arboreal species (2,253) are useful to humans, which represents 84% of the estimated individuals in Amazonian forests. Useful species have mean populations sizes six times larger than non-useful species, and their abundance is related with the probability of usefulness. Incipiently domesticated species are the most abundant. Population size was weakly related to specific uses, but strongly related with the multiplicity of uses. This study highlights the enormous usefulness of Amazonian arboreal species for local peoples. Our findings support the hypothesis that the most abundant plant species have a greater chance to be useful at both local and larger scales, and suggest that although people use the most abundant plants, indigenous people and local communities have contributed to plant abundance through long-term management.

## Introduction

During at least 13,000 years, Amazonian indigenous peoples and local communities have harvested plant products from forests and cultivated numerous species in homegardens, swiddens and agroforests [1]. These plants are used in daily-life, such as food, ornament, poisons, cloths, basketry, medicines and many other uses [2], and some, such as rubber (*Hevea brasiliensis*), have entered national and international markets since European colonization [3]. Different plants have different abundances across landscapes; some tree and palm species (hereafter arboreal species) used by humans are rare [4, 5], while others represent the majority of individuals over large areas, the so-called hyperdominant species [6]. Although relatively few hyperdominant species occur across all of Amazonia, they account together for half of all estimated individual trees and palms in the region [6] and often sustain local livelihoods [7]. Yet, no study has examined the relationship between usefulness of arboreal species and their population sizes at a large scale.

The probability of a plant being adopted into a local culture and becoming used in diverse ways seems to increase when the plants are easily found, gathered and transported to settlements, which is influenced by their abundance and accessibility in the landscape [8]. Among plant uses, different amounts of plant resources (e.g., biomass of leaves, fruits and wood) may be needed according to the given use (e.g., food, housing, medicines), which is also often associated with the species’ abundance in the landscape [9, 10]. However, the hypothesis that people use the most abundant plants is debated, because it is not the only hypothesis that explains the choices of plants for use. The in-depth knowledge people have about plant use, including which plants to choose and how to use and manage them, is the result of a history of reciprocal interactions among people and their environments [11], and leads to another hypothesis: people have increased the abundance of useful plant species through long-term management [12].

During the Holocene, indigenous people transformed Amazonian forests at local and larger scales [13]. Although the abundance of arboreal species is certainly determined by numerous environmental [14] and evolutionary factors [15], the abundance of many useful arboreal species in present-day Amazonian forests may have been enhanced by pre-Columbian people, intentionally or unintentionally, through indigenous management practices [12]. For example, the swidden-fallow sequence often results in resource rich forests [16–18], the Kayapó indigenous peoples of the Xingu River created forest islands in the forest savanna transition [19, 20], and homegardens and diverse polyculture agroforestry systems lead to the cultural forests of the lower Tapajós River basin [21]. These are a few examples that show how simple practices can result in domesticated forest landscapes [22], originally called cultural forests [12], as well as some oligarchic forests [23]. In Amazonian forests, many stands are dominated by one, a few, or multiple useful species [12, 23–25], often domesticated to some degree [22, 26], such as the stands of useful species dominated by the incipiently domesticated Brazil nut (*Bertholletia excelsa*) [27–29] or piquiá (*Caryocar villosum*) [30, 31] near settlements in Central Amazonia.

Here we define plant domestication as a co-evolutionary process of long-term human-plant interactions that starts from the moment that people start selecting, accumulating and caring for plants with desired properties in their ‘*domus*’ [32, 33], and which can lead to visible changes in plant traits of human interest, such as fruit size, color and sweetness [34]. In Amazonia, such management practices are as old as human occupation itself, dating back at least 13,000 years [35]. Hence, plant domestication is a long-term, continuous and open-ended process that extends from populations with incipient changes that are not much different from wild populations to those that depend upon humans for their reproduction and survival [33, 34, 36]. We can classify plant populations during domestication into incipiently-domesticated, semi-domesticated or fully-domesticated populations according to the intensity and duration of these management practices and the morphological and genetic changes they accumulated [33, 34]. The validity of first category is sometimes questioned but is a logical requirement of domestication as a process, since the results of the first selection and cultivation are expected to be smaller than results from subsequent selections [34, 36]. It is important to recognize that not all populations found in Amazonian forests contain individuals with domesticated traits, which is expected in a continental-size area such as Amazonia [26]. Incipiently and semi-domesticated populations can survive and reproduce in forests when abandoned by peoples, such as incipiently domesticated cupuaçu (*Theobroma grandiflorum*) and semi-domesticated cacao (*Theobroma cacao*), respectively, whereas fully-domesticated populations, such as biribá (*Annona mucosa*), depend on human management to survive and reproduce [33, 36]. Of the 85 domesticated arboreal species identified so far in Amazonian forests, fifteen species with incipiently-domesticated populations are hyperdominant, while no species with fully-domesticated populations are hyperdominant [26], which is expected because fully domesticated populations do not survive in old-growth forests [28]. Despite recent advances about the abundance and richness of domesticated species associated with archaeological sites [26], no one has examined the population sizes of species with different degrees of domestication across Amazonia, nor how these compare with useful non-domesticated and non-useful species.

Here we integrate information from ethnobotany and ecology to assess the hypothesis that the most abundant arboreal species have a greater chance to be useful across Amazonia. We classified arboreal species into six use categories (food, medicine, manufacturing, construction, thatching and firewood) and into three degrees of domestication (incipiently, semi and fully-domesticated). We address three major questions: Do abundant species have a higher probability than rare species to be useful? How does population size vary among use categories and among one or multiple use categories? How does population size vary with degree of domestication?

## Methods

### Data collection

We used data of 4,454 arboreal palm and tree species distributed in 1,946 plots across the Amazon basin and Guiana Shield (Amazonia) compiled by the Amazon Tree Diversity Network (ATDN). We started with the data available in ter Steege et al. [6]. We then updated this data set with the currently adopted taxonomic nomenclature of arboreal species and their estimated population sizes for Amazonia available in ter Steege et al. [37], reducing the number of species from 4,962 [6] to 4,454. We used estimated population sizes as a measure of abundance. In the ATDN inventory plots, individuals of trees and palms with ≥ 10 cm DBH were sampled mostly in one-hectare plots located in five main types of mature lowland forests in Amazonia: *terra firme* (non-flooded), white-sand (*campina* and *campinarana*), and seasonally or permanently flooded terrain (*várzea*, *igapó*, swamp).

Ter Steege et al. [6] showed that only 227 hyperdominant arboreal species dominate Amazonian forests and represent half of the total arboreal individuals in Amazonia. After correcting for synonyms [37], 222 species remained for our analysis; their estimated population sizes varied from approximately 298 million to 4.7 billion individuals [for more details of estimated population sizes see ter Steege et al. [37]].

Of the 85 arboreal species known to have domesticated populations [26], we identified 80 in our study (*Annona muricata*, *Annona squamosa*, *Crescentia cujete*, *Elaeis oleifera* and *Sapindus saponaria* were excluded from ATDN datasets after corrections). The list of domesticated species followed Clement et al. [33] and Levis et al. [26]. The degree of domestication was based on magnitude of phenotypic (and sometimes genotypic) variability between cultivated and wild populations following Clement [33] and Levis et al. [26]. Domesticated species were assigned to use categories as with any other useful species.

**Fig 1:**
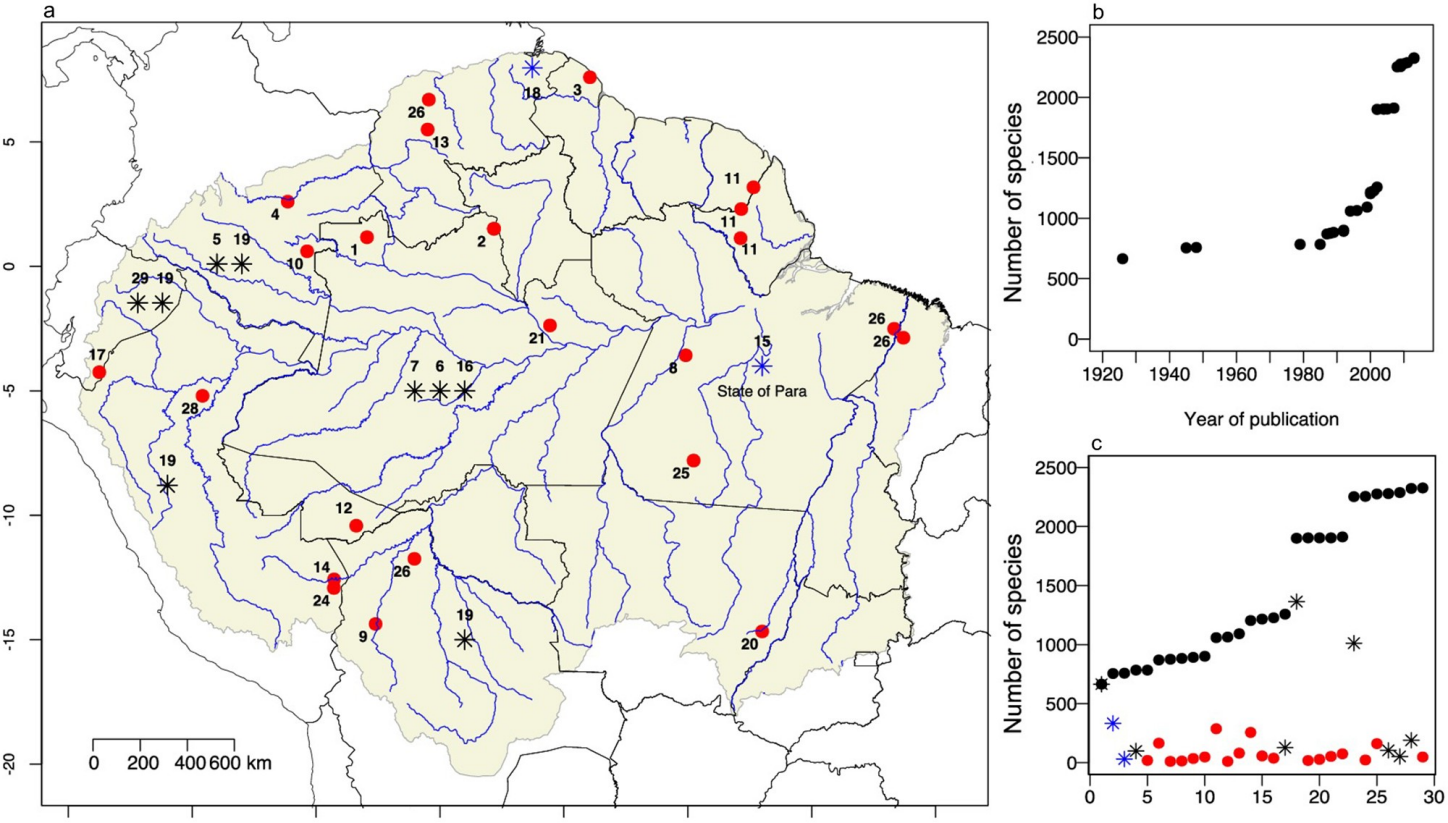
(a) Location of the 29 ethnobotanical studies of useful arboreal species in Amazonia. The citation of the ethnobotanical study (see the corresponding references in S1 Appendix) is given for each location in the map. Ethnobotanical studies are classified in two categories of spatial coverage: local studies (red dots) and compendia (asterisks). Black asterisks represent studies conducted in a country, and blue asterisks represent studies in the State of Pará, Brazil, and the Orinoco River basin, Venezuela. The number of asterisks represents the number of compilations in a given country. The studies of Patiño [38] and Revilla [39] are not represented on the map, since they cover the entire Amazon; (b) Cumulative number of useful arboreal species documented in Amazonia, ordered by the period of publication (1926–2013); (c) Species accumulation curve showing the 29 studies ordered by contribution of new species (black dots) and the total number of useful species that each study contributed to the dataset (red dots, and blue and black asterisks). The highest asterisk values correspond to Corrêa [40], de la Torre et al. [41] and Revilla [39], carried out in Brazil, Ecuador and for all of Amazonia, respectively. Base map source: Natural Earth, Forest-GIS and Eva et al. [42].

We used 29 Amazonian ethnobotanical scientific papers (16), books (9), book chapters (3) and a doctoral dissertation (1) published between 1926 and 2013 in various parts of Amazonia (Fig 1; this reference list is provided in the S1 Text in S1 Appendix) to identify the uses of arboreal species for daily-life or commercial purposes (S1 Table). The ethnobotanical studies covered different regions and ethnic groups, including indigenous people and local communities (Fig 1). Among these, 19 are local studies and 10 are compendia of several studies (Fig 1c and S2 Text in S1 Appendix), including large-scale compilations of local ethnobotanical studies and other large ethnobotanical books and compendia (e.g., the book by Richard E. Schultes, Plants of the Gods). The identification of a use by one culture or community does not mean that all cultures and communities use the species similarly, but does demonstrate a use. We grouped subspecies or varieties mentioned in the studies into the corresponding species, and accepted species with “cf.” identification as belonging to the named species. We only considered studies that adopted botanical nomenclature with specimens identified at the species level and we excluded those that only presented common names.

All uses recorded from the literature review were classified into ethnobotanical categories based on Prance et al. [43] and Macía et al. [44]: food, medicine, manufacturing, construction, thatching and firewood (S1 Table). For each of the arboreal species we assigned a main use category, which was determined by the most cited use category in the references. Assigning a main use may help us have a more comprehensive understanding of what is the most common use for each species. Our final dataset (available in the Data availability section) included the currently adopted taxonomic nomenclature of the species, all use categories mentioned for each species and the references of the plant use information. We constructed a collector curve to assess the cumulative number of useful arboreal species recorded during the last century in the ethnobotanical studies (Fig 1b and 1c). We also performed a literature review of archaeological, archaeobotanical and ecological studies about long-term use of almost all hyperdominant species or genera with incipiently and semi domesticated populations (S2 Table).

### Data analysis

All analyses were performed in R software [45]. To test if useful species tend to be the most abundant species, we compared the mean population sizes of useful and non-useful species with a one-way ANOVA. We also investigated if mean population sizes differ between useful and non-useful species among phylogenetically related species (see S1 Text in S1 Appendix for more details). We used Generalized Linear Mixed Models (GLMM) (lme function of nlme package [46]; S1 Fig), with “Family” and “Genera” as random factors. We adopted this analytical framework to correct for the assumption that more closely related species may have more similar population sizes [15]. We also evaluated the chance that a given species is useful as a function of its population size using a logistic regression (see S1 Text in S1 Appendix for more details). Mean population size was log_10_ transformed before all analyses to normalize variable distribution [47].

To assess whether the hyperdominant non-useful species (species in which the chance to be useful ranges from 81.2 to 93%) are indeed non-useful, we performed a complementary literature search to find at least one use for each of those species (S7 Table in S2 Appendix) in Google Scholar, ScienceDirect and Web of Science, yet we did not use this complementary search in our analysis and results. The search used the scientific name of each plant species with the keywords “plant + use”, “ethnobotanical + studies”, “ethnobotany”, “local + knowledge”.

To assess if population sizes of species differ among the seven use categories, we used bootstraps to estimate means and standard deviations for (i) species used in one use category (called ‘single use’), (ii) species used in more than one use category (‘multiple uses’), and (iii) species that may be used in more than one category, but with a main use category (‘main use category’). The number of use categories varied from zero (no use) to six (all categories). Bootstrapping was applied to account for the large differences in sample sizes (number of species) among the use categories [48].

To assess whether population sizes of species differ among useful and non-useful categories, and the degrees of domestication (incipiently, semi and fully domesticated species), we also performed bootstrap analyses. We identified significant differences by the lack of overlap between standard deviations [49]. For these analyses, we used the function groupwiseMean (R companion package) with a confidence interval of 95% and 9999 randomizations [48]. To understand if larger species population sizes are associated with broader spatial distributions across Amazonia, we evaluated the relationship between the population sizes of species and the number of plots in the ATDN network where they occur across Amazonia using a linear model (LM) after log_10_ transformation to normalize both variables.

## Results

**Fig 2:**
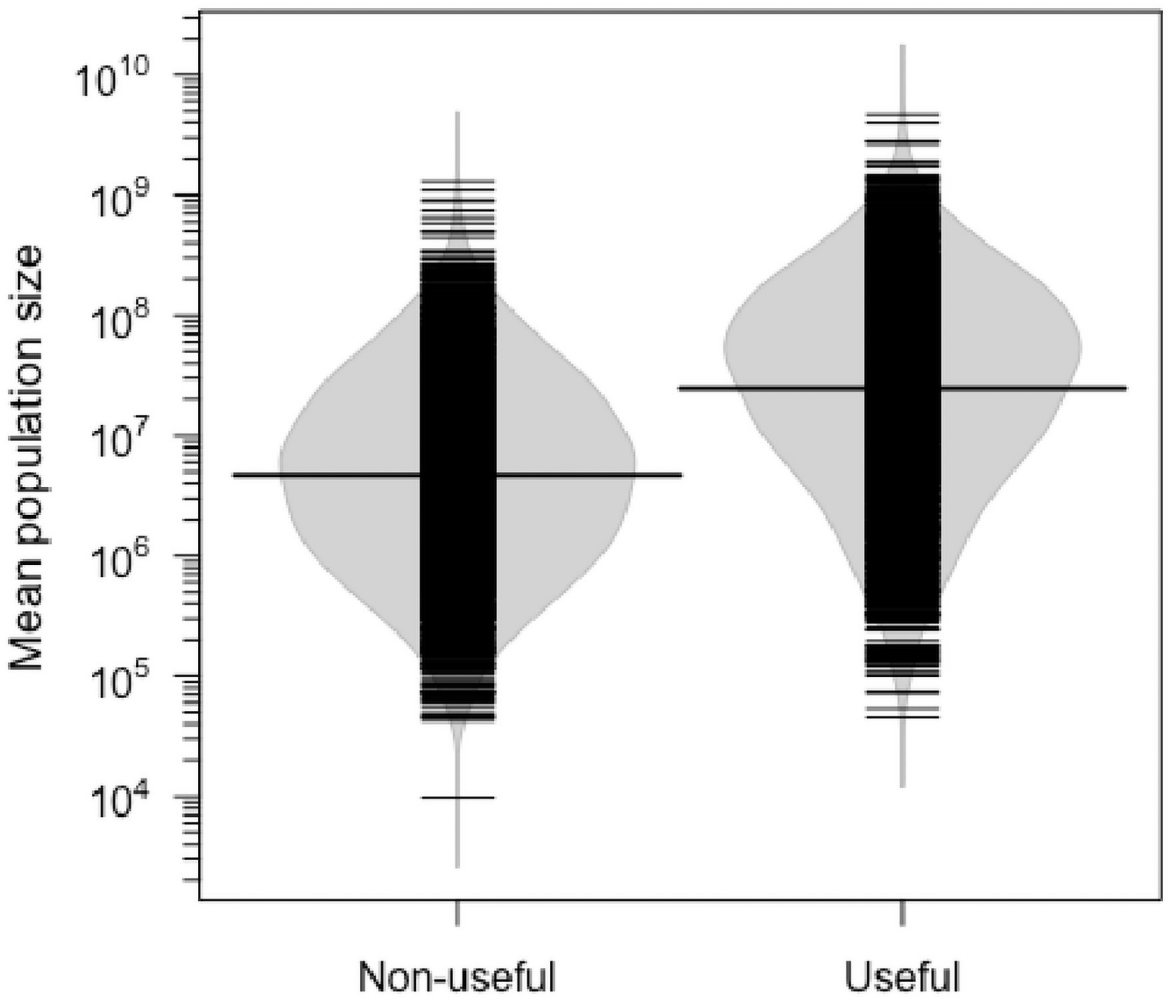
Mean population size of useful and non-useful arboreal species in Amazonia. Small black lines represent the species; large black lines represent the medians; grey shadows represent the density of species. Mean population size was log_10_ transformed (p < 0.01, F = 824.4).

We found that 51% of the arboreal species in the ATDN inventories (2,253 out 4,454) are reported as useful to humans in Amazonia, based on decades of compilations and ethnobotanical studies consulted in this study. Useful species correspond to 84% of the individuals in Amazonian forests (approximately 253 billion out 302 billion individuals. Useful species had a mean estimated population size of 30.8 million individuals, 6.4 times higher than non-useful species (p < 0.01, F = 824.4; Fig 2).

Useful species were also more abundant than non-useful species within genera (p < 0.01; conditional R^2^ = 0.34) and families (p < 0.01; conditional R^2^ = 0.26) (S1 Fig). A large number of genera, 256 out of 701 (36%), are monospecific, 141 of them were useful species and 115 were non-useful species (S1 Fig and see S3 Table in S2 Appendix). Also, a large number of genera that have multiple species (329 out of 445 genera) had both useful and non-useful species. The same pattern between useful and non-useful species was found when the analysis was done by use category within genera (S2 Fig).

We also found that the number of useful arboreal species in Amazonian forests is probably higher than we report in this study (Fig 1b), as the collector curve does not approach an asymptote. The useful species are distributed in 100 of the 113 families (88%) and in 548 of the 701 genera (78%) recorded in the ATDN inventories. Of the 2,253 useful species, 1,569 species are used for construction (70%), 1,037 for food (46%), 1,001 for their medicinal properties (44%), 854 for manufacturing (38%), 302 for firewood (13%) and 46 for thatching (2%). The sum of these percentages exceeds 100 because 1,364 species (61%) have multiple uses. On the other hand, 889 species (39%) are restricted to a single use category: 433 species (19%) are only used for construction, 206 (9%) for food, 178 (7.6%) for their medicinal properties, 60 (2.6%) for manufacturing and 12 (0.5%) only used for firewood. We could not attribute a main use category to 30% of the useful species, which include 21% of the useful hyperdominants [such as murumuru (*Astrocaryum murumuru*) and andiroba (*Carapa guianensis*)], because they presented the same number of citations among two or more use categories. No species has its use restricted to thatching.

**Fig 3:**
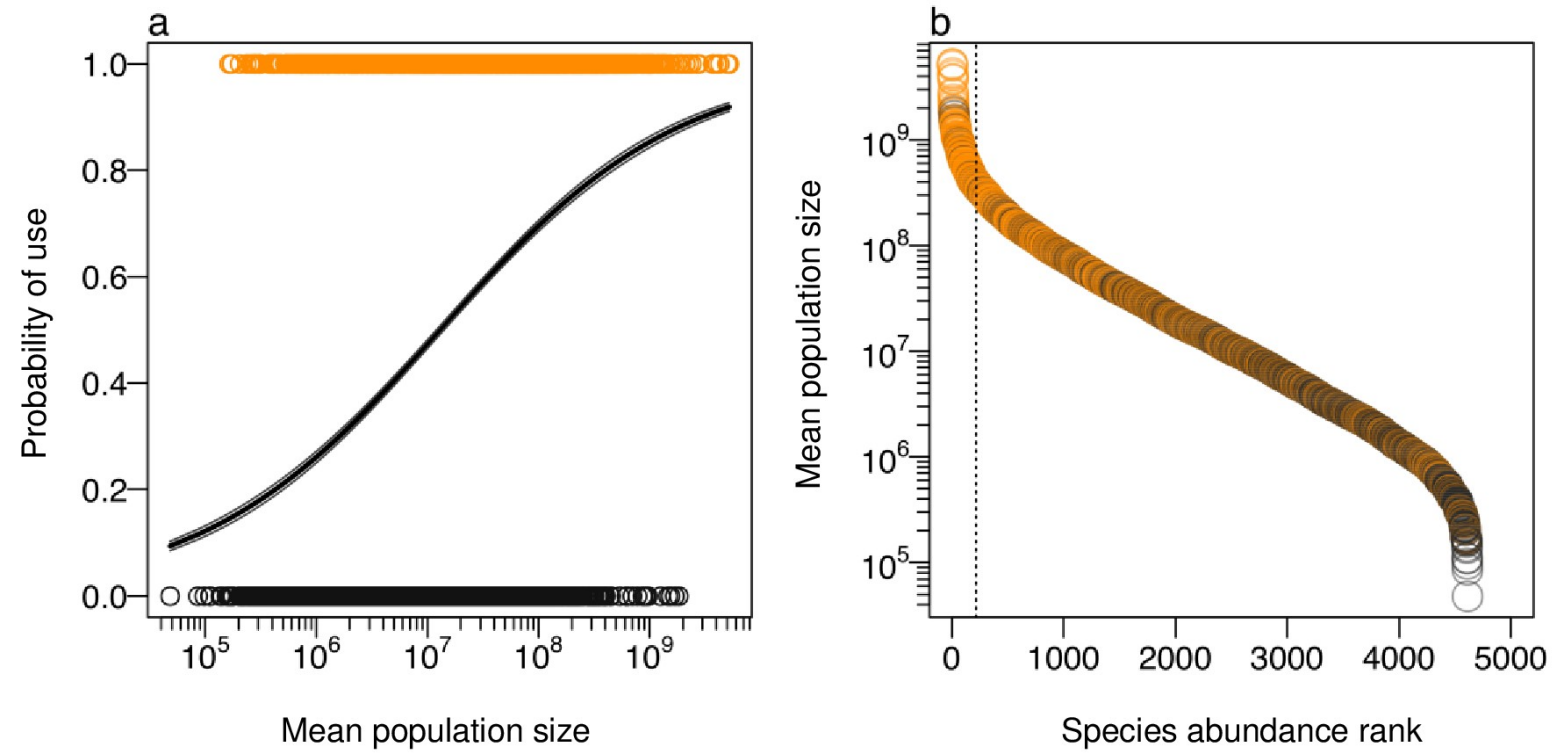
The probability of use and mean population sizes of arboreal species. Orange circles are useful species and black circles are non-useful species. Dashed lines separate hyperdominant and non-hyperdominant species, according to ter Steege et al. [6]. (a) Logistic regression that shows the probability of species being useful according to their mean population sizes (black line); (b) Species abundance rank. Mean population sizes were log_10_ transformed.

The majority of hyperdominant species (207 out 222 species; 93%) are useful (Fig 3a and 3b). The probability for non-hyperdominant species being useful ranges from 4.8% (for the species with the smallest population size) to approximately 81.2%, and the probability of hyperdominant species being useful ranges from 81.2% (for those species with approximately 298 million individuals) to 93% (for the most hyperdominant ones) (Fig 3a). We also investigated if hyperdominant non-useful species are indeed non-useful but found a use for 13 out of the 15 hyperdominant species, previously considered non-useful. We did not find a use for *Eschweilera atropetiolata* and *Pouteria elegans* (S7 Table in S2 Appendix).

**Fig 4:**
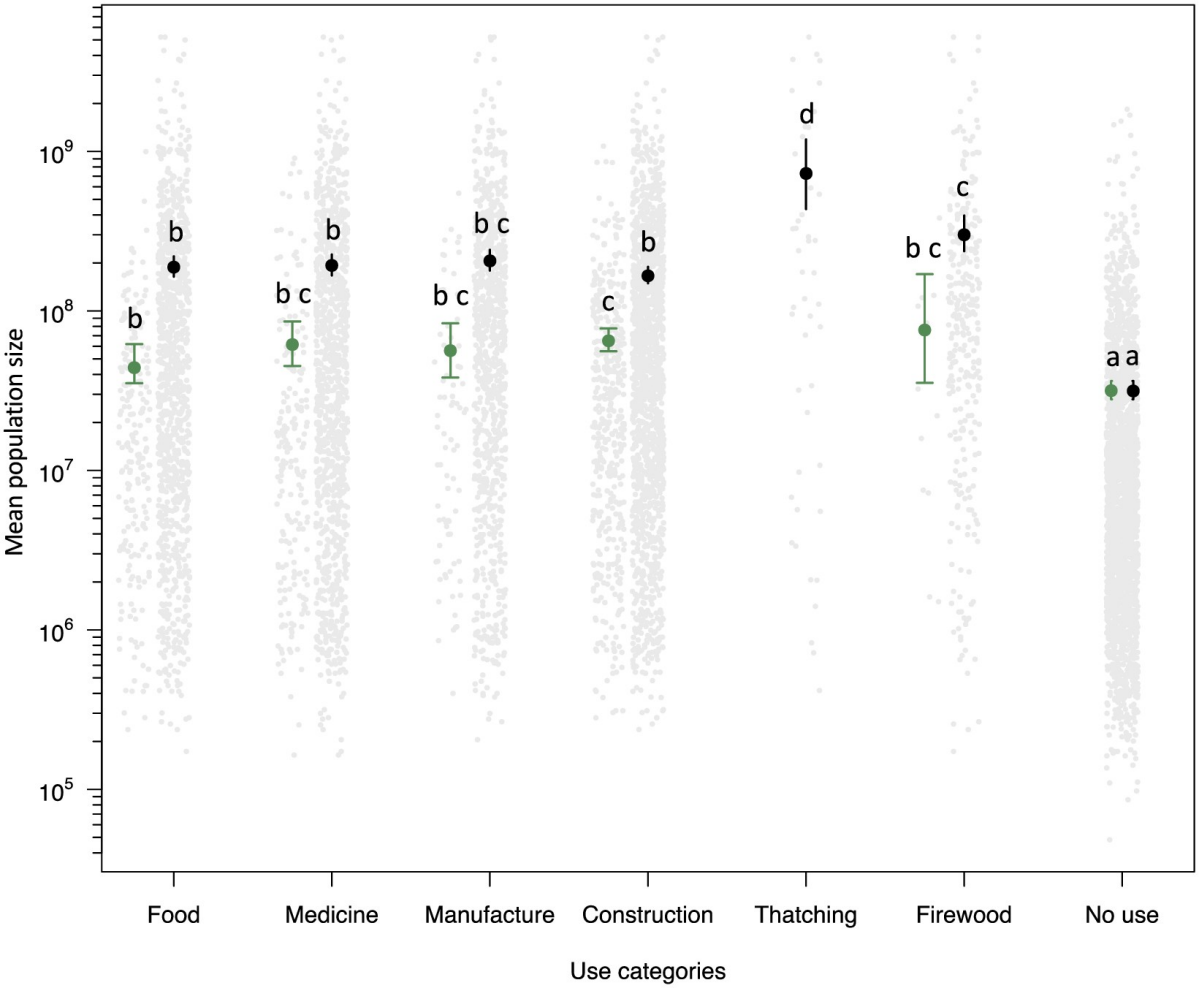
Relationship between the mean population sizes of arboreal species and their use categories. Bootstraps show means and confidence intervals of mean population sizes of species based on their single use (green) and multiple uses (black). The grey points represent each species population sizes. Single use: the species is reported to be used in only one use category. Multiple uses: the species is reported to be used in more than one use category. All thatching species have multiple uses. The bars represent 95% confidence intervals. Mean population sizes were log_10_ transformed. Similar letters above the bars indicate similar mean population sizes.

Useful species exhibited higher mean population sizes in any use category than non-useful species (Fig 4, S3 Fig and S4 Table in S2 Appendix). Looking at the species with multiple uses, population size was the highest for thatching, which only includes the families Arecaceae (19 genera and 45 species) and Lecythidaceae (only *Couratari guianensis*). The firewood category had mean population sizes greater than the food, medicine and construction categories. Species with a single use presented similar mean population sizes among use categories, except for the comparison between food and construction. Food species had mean population sizes smaller than construction species (Fig 4 and S4 Table in S2 Appendix). Species with multiple uses had mean population sizes greater than species with a single use, for all use categories (Fig 4 and S4 Table in S2 Appendix). We also found a positive correlation between mean population size and the number of use categories per species (S4 Fig and S5 Table in S2 Appendix).

**Fig 5:**
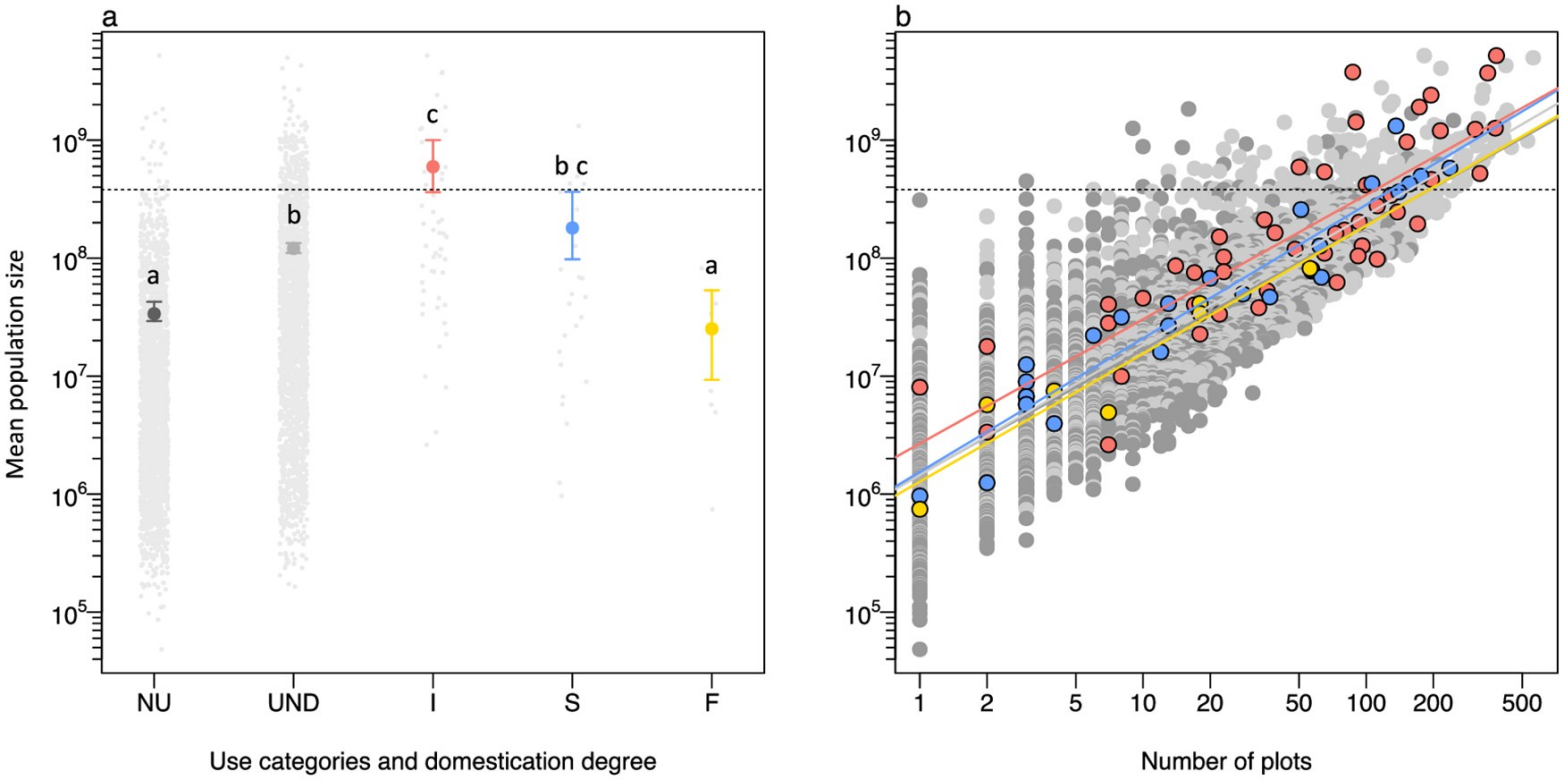
Mean population sizes of species among different scenarios of species’ usefulness and degree of domestication. Non-useful (NU; dark grey), useful non-domesticated (UND; light grey), and incipiently (I; red), semi (S; blue) and fully (F; yellow) domesticated species. Dashed lines separate hyperdominant and non-hyperdominant species. (a) Mean population sizes among the use categories and degrees of domestication. Similar letters above the bars indicate similar mean population sizes; (b) Relationship between mean population size and spatial distribution according to the number of ATDN plots in which the species occurred. The bars represent 95% confidence intervals. Mean population sizes were log_10_ transformed.

Mean population sizes varied among non-useful, useful non-domesticated and domesticated species. Incipiently domesticated species had mean population sizes higher than the fully domesticated, useful non-domesticated species and non-useful species (Fig 5 and S6 Table in S2 Appendix), but similar to semi domesticated species (Fig 5). Fully domesticated and non-useful species have similar and the smallest population sizes in Amazonian forests (see S2 Table for domesticated hyperdominant species).

Strong positive relationships exist between estimated population size and spatial distribution (frequency of ATDN plots with the species) for all species (p < 0.01; R^2^ = 0.79; β = 0.89, Fig 5b), and for each one of the categories: non-useful (p < 0.01; R^2^ = 0.67; β = 0.82); useful non-domesticated (p < 0.01; R^2^ = 0.82; β = 0.91); incipiently domesticated (p < 0.01; R^2^ = 0.80; β = 0.89); semi-domesticated (p < 0.01; R^2^ = 0.91; β = 0.95) and fully domesticated (p < 0.01; R^2^ = 0.82; β = 0.93).

## Discussion

This study expands the scale of the analysis on the uses of Amazonian arboreal species, and sheds light on our understanding about the relationship between present-day abundance of arboreal species and their uses by cultures across Amazonia. These uses are the results of different knowledge and practices, which are dynamic through time and across space, according to the sociocultural and ecological context in which they occur [50]. Together our results reveal that Amazonian forests are dominated by useful arboreal species for many purposes (fibers, shelter, medicine, food, firewood, construction, poisons, dyes, clothes, and many others) and suggest that local people of different cultures often use arboreal species that are more abundant and widespread across Amazonia, not only at the local scale, as shown by previous studies [12, 23, 25]. In addition, the extremely high abundance of incipiently domesticated species among all use and domestication groups suggests that historical domestication processes may have played a role in expanding the dominance of these species in Amazonian forests, in agreement with Levis et al. [26].

### Do abundant species have a higher probability than rare species to be useful?

The population sizes of useful plant species were much higher than those of non-useful species (Fig 2), and include many hyperdominant species, which are more useful than expected by chance. High numbers of individuals of useful plants are commonly observed at local spatial scales in Amazonia [8, 51], including oligarchic forests dominated by useful species, sometimes of commercial importance [22, 23]. These patterns highlight the great usefulness of modern Amazonian forests at scales from the local to the regional, suggesting that the usefulness of a given plant species may be shared among many societies, despite their sociocultural differences. The greater abundance of useful species also holds true within genera and families (S1 and S2 Figs), although closely related species are expected to have similar evolutionary histories and abundances [15]. This implies that the probability of utility increases with abundance across all evolutionary groups despite evolutionary forces that drive patterns of abundance of phylogenetically related species.

Although our model suggests that the probability of a species being useful increases with population size, humans also use rare species (*Annona coriacea* and *Pouteria collina*) (Fig 3). Our finding of rare species being useful may indicate that these species have specific or preferred properties, such as those responsible for edibility or medicinal use [5], or growth form, such as the tall-stemmed palms, used preferentially as materials for souvenirs in northwestern Amazonia irrespective of their abundance [4]. Another possibility for explaining this finding is that many of these species are regionally rare but may be locally abundant, such as *Bactris major* [52] and *Rhizophora mangle* [53].

The opposite pattern is also true; we found that some hyperdominant species are not being used by humans. The existence of two hyperdominant species (*Eschweilera atropetiolata* and *Pouteria elegans*) without a reported use (S7 Table in S2 Appendix) may be explained by several factors, such as: (i) these non-useful species have undesirable traits for people (e.g., fruit taste); (ii) cultural aspects may influence people’s choice and preferences of uses [54]; (iii) uses were not recorded by ethnobotanists [55]; or (iv) we could not find uses for these species in the literature. Lack of data on usefulness may have occurred in our study, although this is less probable for hyperdominant species because they are more easily found in the landscape and their uses have a greater chance to be recorded.

### How does population size vary among use categories and among one or multiple use categories?

Useful species generally had greater population sizes than non-useful species, even if they are used for a particular purpose (Fig 4 and S3 Fig in S2 Appendix), supporting the positive relation between plant use and abundance. Among use categories, the high abundance of species used for thatching is mostly attributed to the use of palms with remarkable economic and cultural importance [56], possibly a reflection of the many uses that most of these species have (on average palm species have four uses while tree species have two uses). Their ancient uses [57] might also have led to an increase of their populations, which is inferred by archaeobotanical studies in Amazonia [58–60]. Contrary to what we expected for those species whose uses involve the extraction of trunks, i.e., suppression of individuals, we found a high overall abundance of species used for manufacturing, construction and firewood. One possible explanation is that physical properties of species for timber and technological uses, such as mechanical resistance or durability, are shared by many species and are more easily substituted by people [5], although there are preferences for some species [61]. Another explanation is that the suppression of individuals locally is probably of low impact (of a few individuals) and does not lead to a decrease in the overall abundance of these species regionally [62], and therefore it is not noticeable in the data used. Moreover, even the cutting of palms and trees by indigenous people may contribute to forest regeneration. The Hi-Merimã people, an isolated Arawá group in the South Amazonas, harvest the fruits of bacaba (*Oenocarpus mapora*), açaí (*Euterpe precatoria*), buriti (*Mauritia flexuosa*), patauá (*Oenocarpus bataua*) and sorveira (*Couma macrocarpa*) by cutting the trees and palms, and the seeds are left in the surroundings of the camps when the individuals are felled [63]. This practice, in addition to the great territorial mobility of Hi-Merimã people, as of other Arawá people, result in an enrichment of useful plants after leaving a territory [63].

Population sizes of arboreal species were positively associated with their number of uses (S4 Fig). This suggests that the wider geographic distribution of the most abundant species might influence the diversity of plant uses due to the diversity of preferences and ethnobotanical knowledge across cultures in Amazonia [43, 44, 54]. Moreover, it is likely that the more uses a species has, the more likely it is to be actively managed because of the benefits gained from this effort [64]. Our results support the idea that exchange networks among indigenous groups in Amazonia [24] may have dispersed many plants with multiple uses [22, 65], such as for food, handicrafts, spiritual purposes and a range of other daily life activities, used not only for utilitarian or economic purposes but also with symbolic and cultural value [66]. Indeed, genetic data have suggested geographical dispersal by people in Amazonia of multi-purpose species, such as Brazil nut [28], peach palm (*Bactris gasipaes*) [67] and cacao (*Theobroma cacao*) [68]. Similar studies for other useful hyperdominant species will allow us to understand better the relation between (past) human populations and the abundance and distribution of arboreal useful species.

### How does population size vary with degree of domestication?

Near archaeological sites, legacies of pre-Columbian and post-Columbian indigenous peoples are detectable in modern forest composition by looking at richness and abundance of domesticated species [26, 69, 70]. Our analysis found that the largest population sizes across Amazonia were generally of incipiently domesticated species (Fig 5). This suggests that incipiently domesticated species were and are managed and propagated by indigenous peoples and local communities, and their populations survive and persist in forests after abandonment and without recent management [26, 70, 71]. Indeed, many incipiently domesticated species are clearly visible in early archaeobotanical records across Amazonia, attesting to ancient food use and probable landscape domestication [57]. Long-term use of almost all hyperdominant species or genera with incipiently domesticated populations have been suggested by archaeological, archaeobotanical and ecological studies (S2 Table) that show an association of past landscape management with the presence of incipiently domesticated species found in archaeological sites, such as the açaí palm (*Euterpe precatoria*), patauá (*Oenocarpus bataua*) and buriti (*Mauritia flexuosa*) [57, 72, 73]. At the Cerro Azul archaeological site in Colombian Amazonia, seeds of inajá (*Attalea maripa*), açaí (*Euterpe precatoria*), buriti (*Mauritia flexuosa*) and patauá (*Oenocarpus bataua*) appear around 12,000 years ago and were probably used for food [74], as they are today. At the Teotônio site in Brazilian Amazon, seed remains of piquiá trees (*Caryocar* sp.) and Brazil nut appear in the early and mid-Holocene associated with the lifeways of these populations [75]. The fact that incipient domesticated species are six times more likely to be hyperdominant than expected by chance suggests that the long-term use of their populations is associated with processes of forest enrichment [22], leaving a lasting legacy in modern Amazonian forests, as previously hypothesized [6].

Modern large-scale economic forces may have also driven changes in population abundance of several commercially important species in Amazonia. During the 20^th^ century, some arboreal species were intensively exploited to supply both local and international markets in Europe and the USA [76]. While some populations of rubber and Brazil nut were enriched through management [27, 70, 77], several other species were depleted by the extraction of individuals through logging [e.g., massaranduba (*Manilkara elata*) and itaúba (*Mezilaurus* spp.) for timber, sorva (*Couma* spp.) for chewing gum and rosewood (*Aniba rosiodora*) for essential oil [3]]. For these species, logging pressure may have reduced their populations near settlements in the last century. Based on projected forest losses, which include historical deforestation from 1900 to future deforestation up to 2050, these species are qualified as globally threatened under IUCN threat status criteria (critically endangered, endangered and vulnerable species) [78].

Fully domesticated populations have low mean population sizes in forests, similar to non-useful species, and do not include any hyperdominant species (Fig 5). This suggests that people do not try to manage these species in mature forests, such as the forests inventoried by the ATDN. Instead, fully domesticated species are adapted and kept in agroecosystems created and maintained by people [22, 34, 79]. A higher number of fully domesticated individuals in Amazonian landscapes is thus expected in homegardens and swiddens where they are still cultivated [33, 34, 36].

### Historical people-plant relationships: Contemporary perspectives

The positive relation between plant use and abundance we found offers information for two currently debated hypotheses [80]: people use the most abundant plants or people have increased plant abundance through long-term management. One of the main hypotheses in ethnobotany is that at the landscape scale the availability of plant species influences their uses due to ease of gathering and transportation [81]. Therefore, the most abundant plant species are more frequently used by people of numerous cultures [82], as observed in this study and across tropical forests in different parts of South America [5, 8, 10, 83]. Although it is reasonable to think that people may have taken advantage of plants that are naturally very abundant due to their adaptation to natural conditions, there is evidence from historical and current studies that indigenous people have influenced the distribution and abundance of useful plants [22, 34]. People’s practices have probably contributed, intentionally or unintentionally, to the enrichment of many useful arboreal species locally [12, 25] and regionally [26]. Yet, questions about the influence of long-term management on the abundance of useful palms and trees in present-day Amazonian forests remain open and more interdisciplinary approaches may help us understand pre-Columbian legacies in Amazonian forests [84].

## Conclusions

Our study reveals that useful arboreal species dominate Amazonian forests at a large scale and highlights the enormous usefulness and socioecological value of these forests to their inhabitants. Our findings of a positive association between arboreal plant uses and their population sizes provides support for the idea that the most abundant species have a greater chance to be useful at local and larger scales. Among use categories, population size was weakly associated with specific uses, yet strongly associated with the number of uses the species have. The extremely high abundance of incipiently domesticated arboreal species and multiple evidences of their ancient use suggest enrichment by past human activities. Given the great abundance of useful and domesticated arboreal species in Amazonian forests, future ecological studies should include human management, as well as environmental and evolutionary factors, to better understand all of the mechanisms involved in the dominance of arboreal species. Our study may help biological conservation science and policies by focusing on conserving sociobiodiversity through the sustainable use of trees and palms in standing forests for the livelihoods and welfare of indigenous and other traditional peoples.

## Supporting information

S1 Appendix (PDF)

S2 Appendix (PDF)

S1 Fig Pairwise comparison of mean population size (log10) between useful and non-useful species within genera and family. (PDF)

S2 Fig Pairwise comparison of mean population size (log10) between useful and non-useful species within genera by use category. (PDF)

S3 Fig Relationship between the mean population sizes of arboreal species and their use categories. (PDF)

S4 Fig Relationship between the mean population sizes of arboreal species and their number of uses, based on the number of use categories the species have. (PDF)

S1 Table Plant uses categories description. (PDF)

S2 Table Hyperdominant species and its ancient uses. (XLSX)
