# Supplementary material for: Eighty-four per cent of all Amazonian arboreal plant individuals are useful to humans
Source: PLoS One. 2021 Oct 1;16(10):e0257875. doi: 10.1371/journal.pone.0257875 (PMC8486103; doi:10.1371/journal.pone.0257875)
Supplement: S1 Appendix — (PDF) [file pone.0257875.s001.pdf]

S1 Appendix

Eighty-four per cent of all Amazonian arboreal plant individuals are useful to humans

Sara D. Coelho*[1], Carolina Levis[1,2,3], Fabrício B. Baccaro[4], Fernando O. G. Figueiredo[5], André Pinassi Antunes[6,7], Hans ter Steege[8,9], Marielos Peña-Claros[2], Charles R. Clement[10], Juliana Schietti[1,4]

[1] Programa de Pós-graduação em Ecologia, Instituto Nacional de Pesquisas da Amazônia, Manaus, AM, Brazil

[2] Forest Ecology and Forest Management Group, Wageningen University & Research, Wageningen, The Netherlands

[3] Programa de Pós-Graduação em Ecologia, Universidade Federal de Santa Catarina, Florianópolis, SC, Brazil

[4] Departamento de Biologia, Universidade Federal do Amazonas, Manaus, AM, Brasil

[5] Coordenação de Biodiversidade, Instituto Nacional de Pesquisas da Amazônia, Manaus, AM, Brazil

[6] RedeFauna - Rede de Pesquisa em Diversidade, Conservação e Uso da Fauna da Amazônia, AM, Brazil

[7] Coordenação de Dinâmica Ambiental, Instituto Nacional de Pesquisas da Amazônia, Manaus, AM, Brazil

[8] Naturalis Biodiversity Center, Leiden, The Netherlands

[9] Systems Ecology, Vrije Universiteit Amsterdam, Amsterdam, The Netherlands

[10] Coordenação de Tecnologia e Inovação, Instituto Nacional de Pesquisas da Amazônia, Manaus, AM, Brazil

* Corresponding author
E-mail: saradeambrozi@gmail.com (SDC)

Text S1: References of the 29 ethnobotanical studies used in the literature review, ordered by their citation number given in this study.

1. Abraão, MB, Shepard, GH, Nelson, BW, Baniwa, JC, Andello, GYDW. Baniwa Vegetation Classification in the White-Sand Campinarana Habitat of the Northwest Amazon, Brazil. In Johnson LM, Hunn ES, editors. Landscape ethnoecology. Concepts of biotic and physical space. New York: Berghahn Books; 2010. pp. 83–115.
2. van Andel TR. Non-timber forest products of the North-West District of Guyana, Part I and II. PhD dissertation, Tropenbos-Guyana Programme. Georgetown, Guyana; 2000. Available from: https://www.tropenbos.org/resources/publications/non-timber+forest+products+of+the+north-west+district+of+guyana+part+i
3. Cárdenas-López D, Canchala N, Arboleda N. Plantas alimenticias no convencionales en Amazonia colombiana y anotaciones sobre otras plantas alimenticias. Revista Colombia Amazónica, Instituto Amazónico de Investigaciones Científicas SINCHI; 2012. pp. 58-81.
4. Couly C, Sist P. Use and knowledge of forest plants among the Ribeirinhos, a traditional Amazonian population. Agroforestry Systems. 2013;87(3):543–554.
5. Corrêa MP. Dicionário das plantas úteis do Brasil e das exóticas cultivadas, 6v. Rio de Janeiro: Imprensa Nacional. 1926-1975.

6. Cavalcante, PB. Frutas comestíveis da Amazônia (7th ed.). Belém: Museu Paraense Emilio Goeldi. 2010.

7. Dewalt SJ, Bourdy G, Michel LRC, Quenevo C. Ethnobotany of the Tacana: quantitative inventories of two permanent plots of Northwestern Bolivia. Economic Botany. 1999;53(3):237–260.

8. Dufour, DL. The composition of some foods used in Northwest Amazonia. Interciência. 1988;13(2): 83–86.

9. Grenand P. The use and cultural significance of the secondary forest among the Wayapi Indians. In: Plotkin M, Famolare L, editors. Sustainable harvest and marketing of rain forest products. Washington, DC: Island Press; 1992. pp. 27-42.

10. Lawrence A, Phillips OL, Ismodes AR, Lopez M, Rose S, Wood D, Farfan AJ. Local values for harvested forest plants in Madre de Dios, Peru: Towards a more contextualised interpretation of quantitative ethnobotanical data. Biodiversity and Conservation. 2005;14(1):45–79.

11. Luna FMS. Ethnobotany of the communities of the upper Rio Nangaritza. Lyonia: A Journal of Ecology and Application. 2004;7(2):105–122.

12. Macía MJ, Armesilla PJ, Cámara-Leret R, Paniagua-Zambrana N, Villalba S, Balslev H, Pardo-de-Santayana M. Palm uses in northwestern South America: a quantitative review. Botanical Review. 2011;77(4):462–570.

13. Marimon BS, Felfili JM. Ethnobotanical comparison of “Pau Brasil” (*Brosimum rubescens* Taub.) forests in a Xavante Indian and a non-Xavante community in eastern Mato Grosso state, Brazil. Economic Botany. 2001;55(4):555–569.

14. Miller RP, Wandelli EV, Grenand P. Conhecimento e utilização da floresta pelos índios Waimiri-Atroari do Rio Camanau-Amazonas. Acta Botanica Brasilica. 1989;3(2):47–56.

15. Patiño VM. Historia y dispersión de los frutales nativos del Neotrópico. Cali, Colombia: Centro Internacional de Agricultura Tropical – CIAT. 2002.

16. Phillips O, Gentry AH, Reynel C, Wilkin P, Galvez-Durand BC. Quantitative ethnobotany and Amazonian conservation. Conservation Biology. 1994;8(1):225–248.

17. Cárdenas-López D, Politis GG. Territorio, movilidad, etnobotánica y manejo del bosque de los Nukak orientales: Amazonía Colombiana (3rd ed.). Colombia: Instituto Amazónico de Investigaciones Científicas SINCHI, Ediciones Uniandes. 2000.

18. Posey DA. Indigenous management of tropical forest ecosystems: the case of the Kayapó Indians of the Brazilian Amazon. Agroforestry Systems. 1985;3:139–158.

19. Prance GT, Balée W, Boom BM, Carneiro RL. Quantitative ethhnobotany and the case for conservation in Amazonia. Conservation Biology. 1987;1(4):296–310.

20. Revilla J. Plantas úteis da bacia Amazônica, 2v. Manaus: Instituto Nacional de Pesquisas da Amazônia/SEBRAE-AM. 2002.

21. López-Zent E, Zent S. Amazonian Indians as ecological disturbance agents: The Hotï of the Sierra de Maigualida, Venezuelan Guayana. In: Carlson TJS, Maffi L, editors. Ethnobotany and Conservation of Biocultural Diversity, Advances in Economic Botany. New York: New York Botanical Garden Press; 2004. pp. 79–112.

22. Smith N, Vásquez R, Wust WH. Frutos del Río Amazonas: Sabores para la conservación. Amazon Conservation Association (ACA): Asociación para la Conservación de la Cuenca Amazónica (ACCA). Lima, Perú: Grafica Biblos S.A. 2007.

23. Loureiro AA, Silva MF, Alencar JC. Essências madeireiras da Amazônia, 2v. Manaus, AM: Instituto Nacional de Pesquisas da Amazônia. 1979.

24. Lowie RH. The tropical forests: An Introduction. In: Steward JH, editors. Handbook of South American Indians. Vol. 3: The tropical forest tribes. Smithsonian Institution, Bureau of American Ethnology, Bulletin 143. Washington, DC: Government Printing Office; 1948. pp. 1-56.

25. Milliken W, Albert B. The use of medicinal plants by the Yanomami Indians of Brazil. Economic Botany. 1996;50(1):10–25.

26. Kainer KA, Duryea ML. Tapping women's knowledge: plant resource use in extractive reserves, Acre, Brazil. Economic Botany. 1992;46(4):408–425.

27. Albert B, Milliken W, Gomez GG. Urihi A: a terra-floresta Yanomami. São Paulo: Instituto Socioambiental; Paris, France: IRD - Institut de Recherche pour le Devéloppment. 2009.

28. de la Torre L, Navarrete H, Muriel P, Marcia M, Balslev H. Enciclopedia de las Plantas Útiles del Ecuador. Quito: Herbario QCA de la Escuela de Ciencias Biológicas de la Pontificia Universidad Católica del Ecuador & Aarhus: Herbario AAU del Departamento de Ciencias Biológicas de la Universidad de Aarhus. 2008.

29. Le Cointe P. O Estado do Pará: a terra, a água e o ar. São Paulo: Companhia Editora Nacional. 1945.

Text S2: Information about the authors of the compendia and large-scale studies used in this research

Cárdenas-López D, Canchala N, Arboleda N. Plantas alimenticias no convencionales en Amazonia colombiana y anotaciones sobre otras plantas alimenticias. Revista Colombia Amazónica, Instituto Amazónico de Investigaciones Científicas SINCHI. 2012; 5: 58-81.

Dairon Cárdenas-López is a biologist. He dedicated his studies to the ecology and flora of Amazonia, including studies of useful plants, endemic plants, forest management, biodiversity conservation and of biological, socioeconomic and cultural diversity of Amazonia. Among many professional experiences, he is also coordinator of the research program in Ecosystems and Natural Resources of the Amazon Scientific Research Institute (Sinchi) and was curator of the Colombian Amazonian herbarium (COAH) (1). The article *Plantas alimenticias no convencionales en Amazonia colombiana y anotaciones sobre otras plantas alimenticias* is based on the information of plant uses across the Colombian Amazon collected from floristic inventories and deposited in herbarium, with emphasis on edible plants.

Cavalcante PB. Frutas comestíveis da Amazônia (7th ed.). Belém: Museu Paraense Emilio Goeldi. 2010.

Paulo Bezerra Cavalcante was a botanist, concentrating his studies on the Amazonian flora and plant taxonomy. Cavalcante worked on the reorganization of the herbarium and the botanical sector of the Emilio Goeldi Museum, Pará, Brazil. He participated in numerous scientific expeditions and floristic surveys in Pará, Amazonas and Amapá, and developed an extensive collection and study of several Amazonian plant species, genera and families (2). The book

Frutas Comestíveis da Amazônia presents edible fruits of Amazonia, their scientific and popular names, synonyms and botanical family.

Corrêa MP. Dicionário das plantas úteis do Brasil e das exóticas cultivadas, 6v. Rio de Janeiro: Imprensa Nacional. 1926-1975.

Manoel Pio Corrêa was a naturalist and botanist. He travelled the world as a researcher dedicating his studies to the forest and floral characteristics, including studies of flora of Brazil and the cultivation of tropical fruits (3). In Brazil, he occupied the position of naturalist in the Botanical Gardens of Rio de Janeiro, member of the Geographic Society of Rio, of Historic and Geographic Institutes, and of the National Museum of Rio, and developed studies on forestry and forest conservation for the department of agriculture (3). As a result of his work, Corrêa published many studies, among which are the six volumes of a large ethnobotanical survey from travels and notes in Brazil, the *Dictionary of Useful Plants of Brazil and of Exotic Cultivated*, published between 1926 and 1975 (volumes 2 and 3 were edited by the Serviço de Informação Agrícola, and volumes 4, 5 and 6 were edited by the Instituto Brasileiro de Desenvolvimento Florestal - IBDF). This book mentions thousands of native and exotic useful plants for subsistence, commercial and industrial uses, their scientific and common names, synonyms, botanical descriptions and illustrations.

Le Cointe P. O Estado do Pará: a terra, a água e o ar. São Paulo: Companhia Editora Nacional. 1945.

Paul Georges Aimé Le Cointe was a French naturalist and lived in Pará, Brazil, through the French Mission. Le Cointe became a chemist and geographer and published many bibliographies

since the beginning of the 1900s about his studies of natural resources of Amazonian flora and their uses (4). He wrote mainly on geography and economy of Amazonia, publishing brochures for the government of Pará of technical materials, such as on cacao, rubber, oilseeds, balsams, resins, rubbers, jutes, balatas and woods from the Amazonian forests (4). The book *O Estado do Pará: a terra, a água e o ar* describes the physical geography of the state of Pará, climate, animals, forests, woods, minerals, with particular emphasis on botany and their applications in industry, food and therapy.

Loureiro AA, Silva MF, Alencar, JC. Essências madeireiras da Amazônia, 2v. Manaus, AM: Instituto Nacional de Pesquisas da Amazônia. 1979.

Arthur A. Loureiro is a researcher, forestry engineer and organizer of the Wood Collection at National Institute of Amazonian Research (INPA) (5). The book *Essências madeireiras da Amazônia* contains technological information of numerous woody forest species of Amazonia. For each woody species described, Loureiro provides their uses, general characteristics of the wood, macroscopic description, physical and mechanical properties, and a glossary of the main terms used in the botanical and anatomical descriptions.

Lowie, R. H. The tropical forests: An Introduction. In: Steward JH, editor. Handbook of South American Indians. Vol 3: The tropical forest tribes. Smithsonian Institution, Bureau of American Ethnology, Bulletin 143. Washington, DC: Government Printing Office; 1948. pp. 1-56.

Robert Harry Lowie was an anthropologist with an ethnological approach and contributed scientifically to ethnological data, such as the translation to English of the Curt Nimuendajú's

manuscripts about some of the least known tribes in eastern Brail, the Ge speaking Indians, that Nimuendajú had visited (6). Lowie was a major contributor to, and editor of, the Tropical Forest volume of the *Handbook of South American Indians* (6), a book with six volumes that systemizes ethnographic studies about the indigenous peoples of South America, including their linguistic, social and historical aspects.

Macía MJ, Armesilla PJ, Cámara-Leret R, Paniagua-Zambrana N, Villalba S, Balslev H, Pardo-de-Santayana M. Palm uses in northwestern South America: a quantitative review. Botanical Review. 2011; 77(4), 462–570.

Manuel J. Macía is a tropical botanist, whose research focuses on understanding the patterns, processes and mechanisms that determine the floristic composition, spatial distribution based on environmental variables, and traditional knowledge of woody-plants in Neotropical rainforests (7). He has studied the patterns of plant use and value by rural and indigenous people, plant-community ecology and relationships between people, plants and habitats. He has also carried out quantitative ethno-botanical and economic botany studies in Ecuador, Bolivia and Mexico (7). In the article *Palm Uses in Northwestern South America: A Quantitative Review*, Macía and collaborators compiled publications of ethnobotanical information for palms occurring in the Amazon and Andes of Colombia, Ecuador, Peru and Bolivia, and the Chocó of Colombia and Ecuador.

Patiño VM. Historia y dispersión de los frutales nativos del Neotrópico. Cali, Colombia: Centro Internacional de Agricultura Tropical - CIAT. 2002.

Víctor Manuel Patiño was an ethnobotanist and dedicated his studies to the traditional knowledge and protection of the natural, agricultural and forestry resources of the Neotropics (8). He is also author of publications on the subjects of agronomy, botany, economic botany, natural history, anthropology and archaeology. Patiño collected species for the germplasm banks of several institutions and worked as an advisor to the Botanic Gardens of several cities in Colombia. In the book *Historia y Dispersion de los Frutales Nativos del Neotrópico*, Patiño identifies and describes the history of the fruiting plants present in the lives of people from the American Ecuadorian region before European conquest.

Revilla J. Plantas úteis da bacia Amazônica, 2v. Manaus: Instituto Nacional de Pesquisas da Amazônia/SEBRAE-AM. 2002.

Juan Revilla is a botanist and conducted numerous studies in economic botany and ethnobotany, including the orientation of the use of Amazonian plants of medicinal value in the treatment for many diseases (9). Revilla published many scientific studies in national and international journals about the Amazonian flora. In the book *Plantas úteis da bacia Amazônica*, Revilla compiled information of thousands of plant uses throughout the Amazon basin.

de la Torre L, Navarrete H, Muriel P, Macía MJ, Balslev H. Enciclopedia de las Plantas Útiles del Ecuador. Quito: Herbario QCA de la Escuela de Ciencias Biológicas de la Pontífica Universidad Católica del Ecuador & Aarhus: Herbario AAU del Departameto de Ciencias Biológicas de la Universidad de Aarhus; 2008. 956 p.

Lucía de la Torre is an ethnobotanist specialized in biodiversity informatics and web-based presentation of data about useful plants (10). In addition to other ethnoecological studies, she has

worked on the relative importance of socioeconomic and ecological factors in determining plant use patterns in Ecuador (10). In the book *Enciclopedia de las Plantas Útiles del Ecuador,* de la Torre and collaborators include a catalogue of more than 5,000 useful plants from Ecuador, with information compiled from ethnobotanical publications and herbarium collections.

References

1. Gobierno de Colombia. Plataforma SCIENTI [cited May 2021]. Available from: http://scienti.colciencias.gov.co:8081/cvlac/visualizador/generarCurriculoCv.do?cod_rh=0000153583
2. Secco R. Em memória de Paulo Bezerra Cavalcante (1922-2006). Bol Mus Para Emílio Goeldi Ciências Naturais, Belém. 2006;1(1):189–90.
3. Prominent in Pan American Affairs. In: Bulletin of the Pan American Union. vol. XXXV. Union of American Republics; 1912. p. 343–50.
4. Meirelles Filho J. Grandes Expedições à Amazônia Brasileira, vol II, Século XX. São Paulo: Metalivros; 2011. 241 p.
5. Loureiro AA, Silva MF, Alencar J da C. Essências madeireiras da Amazônia 2v. Manaus-AM: INPA; 1979.
6. Steward JH. Robert Harry Lowie. A Biographical Memoir. National Academy of Sciences. Washington, D.C.: National Academy of Sciences; 1974.
7. Macía MJ. Manuel J. Macía [cited April 2021]. Research Gate. Available from: https://www.researchgate.net/profile/Manuel-Macia-3
8. Patiño VM. Historia y dispersión de los frutales nativos del Neotrópico. Cali, Colombia: Centro Internacional de Agricultura Tropical - CIAT; 2002. 655 p.

9. Ascom Inpa. Pesquisas e orientações com ervas medicinais rendem a Juan Revilla o Troféu Profissionais do Ano [cited May 2021]. Amazonas Notícias. 2016. Available from: https://amazonasnoticias.com.br/pesquisas-e-orientacoes-com-ervas-medicinais-rendem-a- juan-revilla-o-trofeu-profissionais-do-ano/

10. de la Torre L. Lucia de la Torre [cited April 2021]. Palm Harvest Impacts in Tropical Forests. Use of natural resources: The impact on biodiversity, ecosystem, goods and services. EU Seventh Framework Programme. Available from: https://fp7-palms.au.dk/index.php/participants/pontificia-universidad-catolica-de-ecuador/lucia-de-la-torre.html
