# Supplementary material for: Eighty-four per cent of all Amazonian arboreal plant individuals are useful to humans
Source: PLoS One. 2021 Oct 1;16(10):e0257875. doi: 10.1371/journal.pone.0257875 (PMC8486103; doi:10.1371/journal.pone.0257875)
Supplement: S2 Appendix — (PDF) [file pone.0257875.s002.pdf]

S2 Appendix

Eighty-four per cent of all Amazonian arboreal plant individuals are useful to humans

Sara D. Coelho*[1], Carolina Levis[1,2,3], Fabrício B. Baccaro[4], Fernando O. G. Figueiredo[5], André Pinassi Antunes[6,7], Hans ter Steege[8,9], Marielos Peña-Claros[2], Charles R. Clement[10], Juliana Schietti[1,4]

[1] Programa de Pós-graduação em Ecologia, Instituto Nacional de Pesquisas da Amazônia, Manaus, AM, Brazil

[2] Forest Ecology and Forest Management Group, Wageningen University & Research, Wageningen, The Netherlands

[3] Programa de Pós-Graduação em Ecologia, Universidade Federal de Santa Catarina, Florianópolis, SC, Brazil

[4] Departamento de Biologia, Universidade Federal do Amazonas, Manaus, AM, Brasil

[5] Coordenação de Biodiversidade, Instituto Nacional de Pesquisas da Amazônia, Manaus, AM, Brazil

[6] RedeFauna - Rede de Pesquisa em Diversidade, Conservação e Uso da Fauna da Amazônia, AM, Brazil

[7] Coordenação de Dinâmica Ambiental, Instituto Nacional de Pesquisas da Amazônia, Manaus, AM, Brazil

[8] Naturalis Biodiversity Center, Leiden, The Netherlands

[9] Systems Ecology, Vrije Universiteit Amsterdam, Amsterdam, The Netherlands

[10] Coordenação de Tecnologia e Inovação, Instituto Nacional de Pesquisas da Amazônia, Manaus, AM, Brazil

* Corresponding author
E-mail: saradeambrozi@gmail.com (SDC)

Text S3: Phylogenetically related species

We only compared mean population sizes of those genera and families that contain both useful and non-useful species. To control for phylogenetic correlations among species, we defined “genus” or “family” as random factors in the analyses. We estimated the separate contributions of both fixed effects (category of use) and random effects (genera or family) calculating the marginal and conditional R^2 of each model. Marginal R^2 identifies the variation explained only by the fixed effect, while conditional R^2 identifies the variation explained by fixed and random effects in the model (1). For all GLMM analyses, we tested the random intercept model and the random intercept and slope model, and choose those with the lowest AIC value (2). We repeated this analytical sequence for each use category and for the subset of genera with domesticated species. We also created a GLM with binomial error distribution to predict the probability of species being useful according to their population size. We used $\log_{10}$ transformations before the analysis to normalize the mean population sizes.

Table S3. Genera and family names included in the pairwise comparison between useful and non-useful species. (A) Genera that have both useful and non-useful species; (B) only genera with useful species and only genera with non-useful species; (C) families with both useful and non-useful species and (D) only genera that have domesticated species and non-useful species.

A	All genera were included, except genera listed in B
B	Genera that have only useful species:<br><br>Abuta, Acanthosyris, Achatocarpus, Acrocomia, Adiscanthus, Aiphanes, Alchorneopsis, Allantoma, Amaioua, Ambelania, Amburana, Ampelocera, Amphiodon, Amphirrhox, Anadenanthera, Anaueria, Antrocaryon, Aparisthmium, Aphandra, Aptandra, Apuleia, Attalea, Bactris, Bagassa, Balizia, Batesia, Batocarpus, Bertholletia, Bertiera, Bixa, Bocoa, Bothriospora, Bribria, Brunfelsia, Cabralea, Calatola, Callisthene, Capirona, Carapa, Caryocar, Caryodaphnopsis, Caryodendron, Casimirella, Castilla, Cavanillesia, Cedrela, Cedrelinga, Centrolobium, Cespedesia, Chelyocarpus, Chlorocardium, Chloroleucon, Chromolucuma, Clarisia, Clavija, Cochlospermum, Cojoba, Combretum, Commiphora, Compsoneura, Condaminea, Conostegia, Couma, Couroupita, Coutarea, Crateva, Curatella, Curupira, Cybistax, Dalbergia, Damburneya, Dialium, Diclinanona, Dicorynia, Dictyocaryum, Dictyoloma, Dicypellium, Didymocistus, Dilodendron, Dinizia, Discophora, Duckeodendron, Duckesia, Dystovomita, Endopleura, Euceraea, Euxylophora, Fusaea, Gallesia, Genipa, Glycydendron, Goupia, Grias, Guazuma, Guettarda, Haploclathra, Hasseltia, Haydenia, Helicostylis, Heliocarpus, Hernandia, Herrania, Holocalyx, Huberodendron, Huertea, Hura, Hydrangea, Iriartea, Iriartella, Jacaranda, Jacaratia, Joannesia, Lafoensia, Laplacea, Lecointea, Leonia, Leopoldinia, Libidibia, Lunania, Macbrideina, Maclura, Macoubea, Magonia, Maieta, Manicaria, Manihot, Maprounea, Maquira, Margaritaria, Mauritia, Mauritiella, Metteniusa, Micrandropsis, Microdesmia, Minquartia, Mucoa, Myriocarpa, Myrocarpus, Myroxylon, Nealchornea, Neocouma, Ochroma, Oenocarpus, Ophiocaryon, Osteophloeum, Otoba, Parachimarrhis, Paramachaerium, Parapiptadenia, Parinariopsis, Patinoa, Pentaclethra, Pentagonia, Pentaplaris, Peridiscus, Physocalymma, Phytelephas, Picrolemma, Plathymenia, Platonia, Platypodium, Pleuranthodendron, Poeppigia, Pogonophora, Poraqueiba, Potalia, Poulsenia, Prunus, Pseudima, Pseudolmedia, Pseudomalmea, Pseudosenefeldera, Psidium, Pterogyne, Ptychopetalum, Raphia, Raputia , Rhamnidium, Rhigospira, Rhodothyrsus, Richeria, Ruagea, Ruizodendron, Sacoglottis, Salvertia, Sambucus, Sarcaulus, Schinopsis, Schizolobium, Scleronema, Semaphyllanthe, Sideroxylon, Simarouba, Socratea, Sohnreyia, Sparattosperma, Spondias, Spongiosperma,

	Stephanopodium, Stylogyne, Swietenia, Syagrus, Symmeria, Symphonia, Tessmannianthus, Tetrastylidium, Tetrathylacium, Theobroma, Ticorea, Tocoyena, Tovomitidium, Trema, Trichanthera, Trichostigma, Trophis, Turpinia, Ximenia, Zeyheria, Ziziphus<br><br>Genera that have only non-useful species:<br><br>Acanthocladus, Acosmium, Actinostemon, Adelia, Amyris, Angostura, Anomalocalyx, Antonia, Archytaea, Ateleia, Ayenia, Barnebydendron, Bathysa, Billia, Blastemanthus, Blepharocalyx, Bonyunia, Bursera, Candolleodendron, Carapichea, Cardiopetalum, Centronia, Chaunochiton, Chionanthus, Chomelia, Ciliosemina, Cinchonopsis, Cinnamodendron, Cleidion, Clerodendrum, Clusiella, Conchocarpus, Cosmibuena, Coursetia, Cyclolobium, Cymbopetalum, Cyrilla, Cyrillopsis, Dahlstedtia, Daphnopsis, Dialypetalanthus, Dichapetalum, Dicymbe, Digomphia, Diplokeleba, Diploon, Diptychandra, Discocarpus, Elaeodendron, Elizabetha, Elvasia, Eumachia, Euphronia, Exostema, Exostyles, Fissicalyx, Froesia, Froesiodendron, Fusispermum, Geissanthus, Glandonia, Guianodendron, Hebepetalum, Helianthostylis, Homalium, Hortia, Hylocarpa, Hyperbaena, Jacqueshuberia, Joosia, Kielmeyera, Ladenbergia, Laxoplumeria, Lepidocordia, Lissocarpa, Loxopterygium, Lozania, Luetzelburgia, Maburea, Macrocnemum, Magnolia, Mahurea, Malmea, Margaritopsis, Melicoccus, Meriania, Metrodorea, Muellera, Myracrodruon, Myrceugenia, Myrcianthes, Neocalyptrocalyx, Neoptychocarpus, Ochthocosmus, Paloue, Paradrypetes, Pentascyphus, Phyllostylon, Pilocarpus, Piptocoma, Pisonia, Platycarpum, Pleradenophora, Plumeria, Pochota, Podocalyx, Podocarpus, Poecilanthe, Pogonopus, Poincianella, Porocystis, Prockia, Pseudomonotes, Pterandra, Pterygota, Raputiarana, Rauia, Recordoxylon, Retiniphyllum, Rinoreocarpus, Ronabea, Rosenbergiodendron, Ruptiliocarpon, Rustia, Schistostemon, Schoepfia, Scyphonychium, Sebastiania, Seguieria, Senefeldera, Senefelderopsis, Siphoneugena, Spachea, Sphinctanthus, Spiranthera, Steinbachiella, Stenostomum, Styloceras, Suessenguthia, Sweetia, Systemonodaphne, Syzygium, Tepuianthus, Touroulia, Varronia, Vaupesia, Votomita, Vouarana, Wallacea, Williamodendron, Wittmackanthus, Yasunia, Zapoteca
C	All families were included
D	Acioa, Acrocomia, Alibertia, Anacardium, Anadenanthera, Annona, Astrocaryum, Attalea, Bactris, Bertholletia, Bixa, Brosimum, Byrsonima, Campomanesia, Campsiandra, Caryocar, Caryodendron, Cassia, Chrysophyllum, Couepia, Couma, Deguelia, Dipteryx, Erisma, Eugenia, Euterpe, Garcinia, Genipa, Grias, Hevea, Hymenaea, Ilex, Inga, Lecythis, Macoubea, Manilkara, Matisia, Mauritia, Myroxylon, Oenocarpus, Phytelephas, Platonia, Poraqueiba, Pourouma, Pouteria, Psidium, Spondias, Sterculia, Talisia, Theobroma, Trema

Table S4. Mean population size and lower (CI lower) and upper (CI upper) confidence intervals values (95 %) of the use categories. Similar letters indicate that means do not differ between the use categories.

	**Use categories**	**Mean**	**CI lower**	**CI Upper**
**Most cited use category**	Food	14.2e+07 a	11.8e+07	17.5e+07
	Medicine	8.99e+07 a	7.03e+07	12.0e+07
	Manufacture	12.2e+07 a	8.65e+07	17.8e+07
	Construction	11.9e+07 a	10.3e+07	14.6e+07
	Firewood	9.80e+07 a	5.30e+07	17.7e+07
	No use	2.23e+07 c	1.99e+07	2.56e+07
**Multiple use categories**	Food	1.52e+08 b	1.34e+08	1.76e+08
	Medicine	1.63e+08 b	1.44e+08	1.88e+08
	Manufacture	1.75e+08 b c	1.54e+08	2.04e+08
	Construction	1.38e+08 b	1.25e+08	1.55e+08
	Thatching	5.48e+08 d	3.45e+08	8.73e+08
	Firewood	2.39e+08 c	1.94e+08	3.04e+08
	No use	0.223e+08 a	0.199e+08	0.256e+08
**Single use categories**	Food	3.54e+07 b	2.92e+07	4.50e+07
	Medicine	5.42e+07 b c	3.98e+07	7.48e+07
	Manufacture	3.81e+07 b c	2.60e+07	5.52e+07
	Construction	5.91e+07 c	4.93e+07	7.38e+07
	Firewood	5.28e+07 b c	2.77e+07	11.1e+07
	No use	2.23e+07 a	1.99e+07	2.56e+07

Table S5. Mean population size and lower (CI lower) and upper (CI upper) confidence intervals values (95 %) of the use categories. Similar letters indicate that means do not differ between the number of use categories the species have.

**Number of use categories**	**N species**	**Mean**	**CI lower**	**CI Upper**
0 use category (no use)	2201	2.23e+07 a	2.00e+07	2.55e+07
1 use category	890	5.11e+07 b	4.51e+07	5.90e+07
2 use categories	611	7.68e+07 c	6.78e+07	8.84e+07
3 use categories	421	14.5e+07 d	12.6e+07	16.7e+07
4 use categories	239	23.7e+07 e	19.5e+07	31.1e+07
5 use categories	75	30.8e+07 e	22.4e+07	44.3e+07
6 use categories	17	119.0e+07 f	73.3e+07	181.0e+07

Table S6. Mean population size and lower (CI lower) and upper (CI upper) confidence intervals (95 %) values of the non-useful, useful non-domesticated and domesticated species. Similar letters indicate that means do not differ between the use categories.

Categories	Mean	CI lower	CI Upper
Non-useful	0.223e+08 a	0.200e+08	0.255e+08
Useful non-domesticated	1.04e+08 b	0.959e+08	1.15e+08
Incipient	4.52e+08 c	2.87e+08	7.55e+08
Semi	1.60e+08 b c	0.844e+08	3.04e+08
Full	0.476e+08 a	0.0893e+08	0.869e+08

Table S7. List of uses of the 15 hyperdominant arboreal species before classified as non-useful species and predicted to be useful (81.2 % to 93 % chance) according to our model. The botanical name of the species, the use categories of the species and the references from the literature review were described in this table. Use categories: (Fo) Food, (Me) Medicine, (Ma) Manufacturing, (Co) Construction, (T) Thatching and (Fw) Firewood.

Species	Useful	Use category	References
Alchornea discolor	yes	Ma	(3)
Eperua leucantha	yes	Me	(4)
Eschweilera atropetiolata	not found		
Eschweilera pedicellata	yes	Ma	(5)
Lueheopsis rosea	yes	Fo	(6)
Metrodorea flavida	yes	Fo, Ma, Co, Fw	(7)
Micrandra sprucei	yes	Fo	(8)
Ocotea cinerea	yes	Me, Ma, Co	(9)
Oxandra polyantha	yes	Co	(10)
Pouteria elegans	not found		
Protium apiculatum	yes	Me, Co, Fw	(11)
Quararibea ochrocalyx	yes	Fo	(12)
Rinorea guianensis	yes	Me, Fw	(7)
Rinoreocarpus ulei	yes	Me, Ma, Co, Fw	(7)
Sagotia brachysepala	yes	Me	(13)

References

1 Nakagawa S, Schielzeth H. A general and simple method for obtaining R2 from generalized linear mixed-effects models. Methods Ecol Evol. 2013;4(2):133–42.

2. Zuur AF, Ieno EN, Smith GM, Saveliev AA, Walker N. Mixed Effects Models and Extensions in Ecology with R. New York: Springer; 2009.

3. Wittmann F, Wittmann AO. Use of Amazonian Floodplain Trees. In: Junk W, Piedade M, Wittmann F, Schöngart J, Parolin P, editors. Amazonian Floodplain Forests: Ecophysiology, Biodiversity and Sustainable Management. New York, London: Springer; 2010. p. 389–418.

4. Baker M, Neill D, Palacios W, Zaruma J. Plant Resources of Amazonian Ecuador. Flora del Ecuador. In: Second Annual Report. Missouri Botanical Garden and New York Botanical Garden; 1987. p. 449–527.

5. Alarcón JGS, Peixoto AL. Use of terra firme forest by Caicubi caboclos, middle Rio Negro, Amazonas, Brazil. A quantitative study. Econ Bot. 2008;62(1):60–73.

6. Ferreira MJ, Levis C, Iriarte J, Clement CR. Legacies of intensive management in forests around pre-columbian and modern settlements in the Madeira-Tapajós interfluve, Amazonia. Acta Bot Brasilica. 2019;1–9.

7. Paniagua-Zambrana NY, Bussmann RW, Hart RE, Moya Huanca AL, Ortiz Soria G, Ortiz Vaca M, et al. Traditional knowledge hiding in plain sight - twenty-first century ethnobotany of the Chácobo in Beni, Bolivia. J Ethnobiol Ethnomed. 2017;13(1):1–47.

8. Schultes RE. Diversas plantas comestíveis nativas do noroeste da Amazonia. Acta Amaz. 1977;7(3):317–27.

9. Odonne G, Bel M, Burst M, Brunaux O, Bruno M, Dambrine E, et al. Long-term influence of early human occupations on current forests of the Guiana Shield. Ecology. 2019;100(10):1–14.

10. Sánchez M, Duivenvoorden JF, Duque A, Miraña P, Cavelier J. A stem-based ethnobotanical quantification of potential rain forest use by Mirañas in NW Amazonia. Ethnobot Res Appl. 2005;3:215–29.

11. Marín-Corba C, Cárdenas-López D, Suárez-Suárez S. Use Value usefulness in ethnobotany. Case study in Putumayo department (Colombia). Caldasia. 2005;27(1):89–101.

12. Ducke J, Martinez R. Amazonian Ethnobotanical Dictionary. 1st ed. Raton, Florida: CRC Press; 1994. 224 p.

13. Kffuri CW, Lopes MA, Ming LC, Odonne G, Kinupp VF. Antimalarial plants used by indigenous people of the Upper Rio Negro in Amazonas, Brazil. J Ethnopharmacol. 2016;178:188–98.
