# Supplementary material for: Eighty-four per cent of all Amazonian arboreal plant individuals are useful to humans
Source: PLoS One. 2021 Oct 1;16(10):e0257875. doi: 10.1371/journal.pone.0257875 (PMC8486103; doi:10.1371/journal.pone.0257875)
Supplement: S1 Fig — (PDF) [file pone.0257875.s003.pdf]

S1 Fig. Pairwise comparison of mean population size (log_{10}) between useful and non-useful species within genera and family. (a) Genera that have both useful and non-useful species; (b) only genera with useful species and genera with non-useful species; (c) families with both useful and non-useful species and (d) only genera that have domesticated (useful) species and non-useful species. At the level of genera, *use* accounted for 14 % of the variation in population size (marginal $R^2 = 0.14$), while *use* and *genus* together explained 34 % (conditional $R^2 = 0.34$; $\beta = 0.64$). When we analyzed only genera containing domesticated species, *use* accounted for 14% of the variance (marginal $R^2 = 0.14$), while *use* and *genus* together explained 35% (conditional $R^2 = 0.35$; $\beta = 0.65$) (for genera and family names included in the pairwise comparison see S3 Table in S2 Appendix).

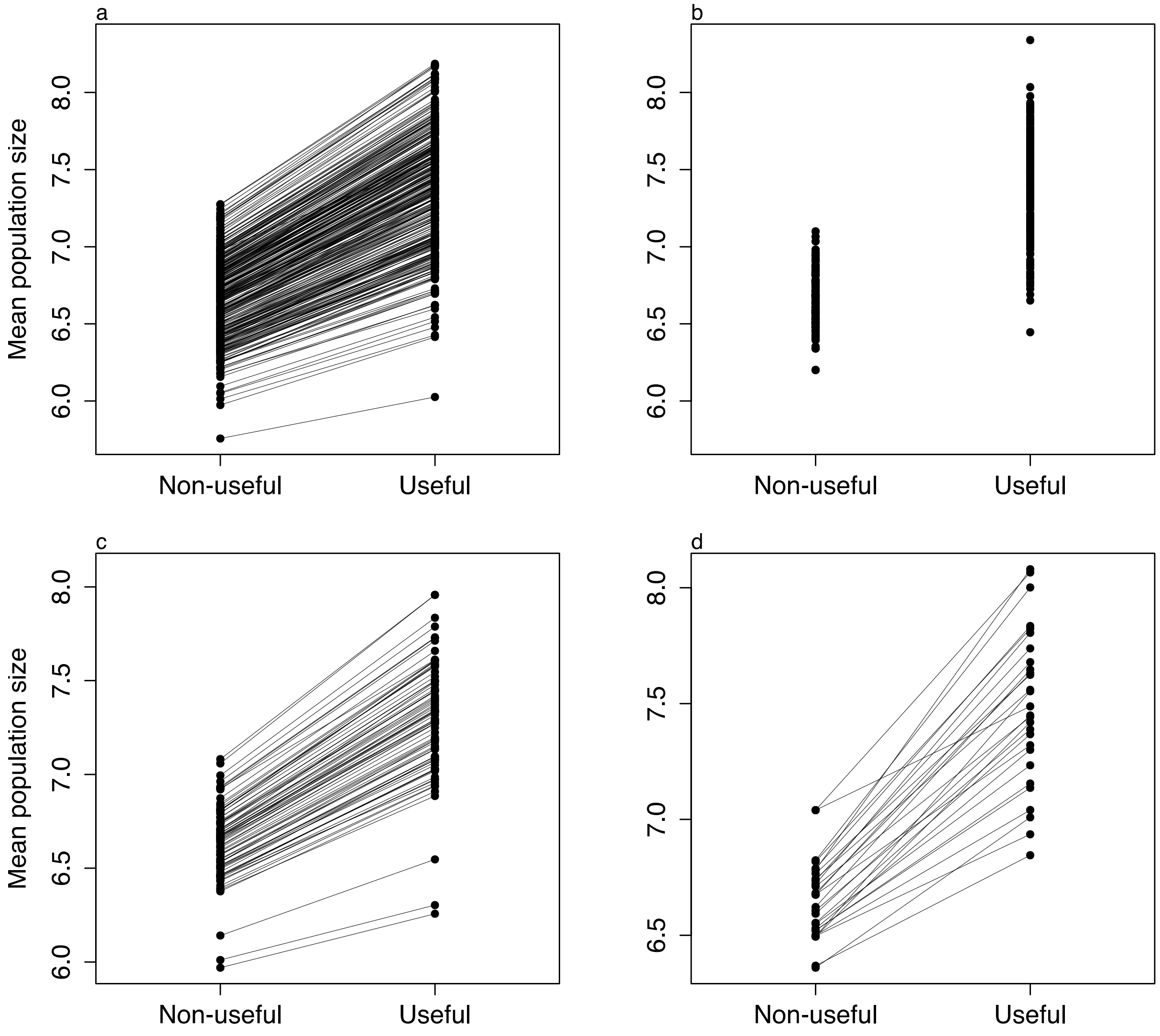
a
b
c
d
Mean population size
Mean population size
6.0
6.5
7.0
7.5
8.0
Non-useful
Useful
