# Supplementary material for: Eighty-four per cent of all Amazonian arboreal plant individuals are useful to humans
Source: PLoS One. 2021 Oct 1;16(10):e0257875. doi: 10.1371/journal.pone.0257875 (PMC8486103; doi:10.1371/journal.pone.0257875)

S2 Fig. Pairwise comparison of mean population size ($\log_{10}$) between useful and non-useful species within genera by use category: (a) food; (b) medicine; (c) manufacturing; (d) construction; (e) firewood and (f) thatching.

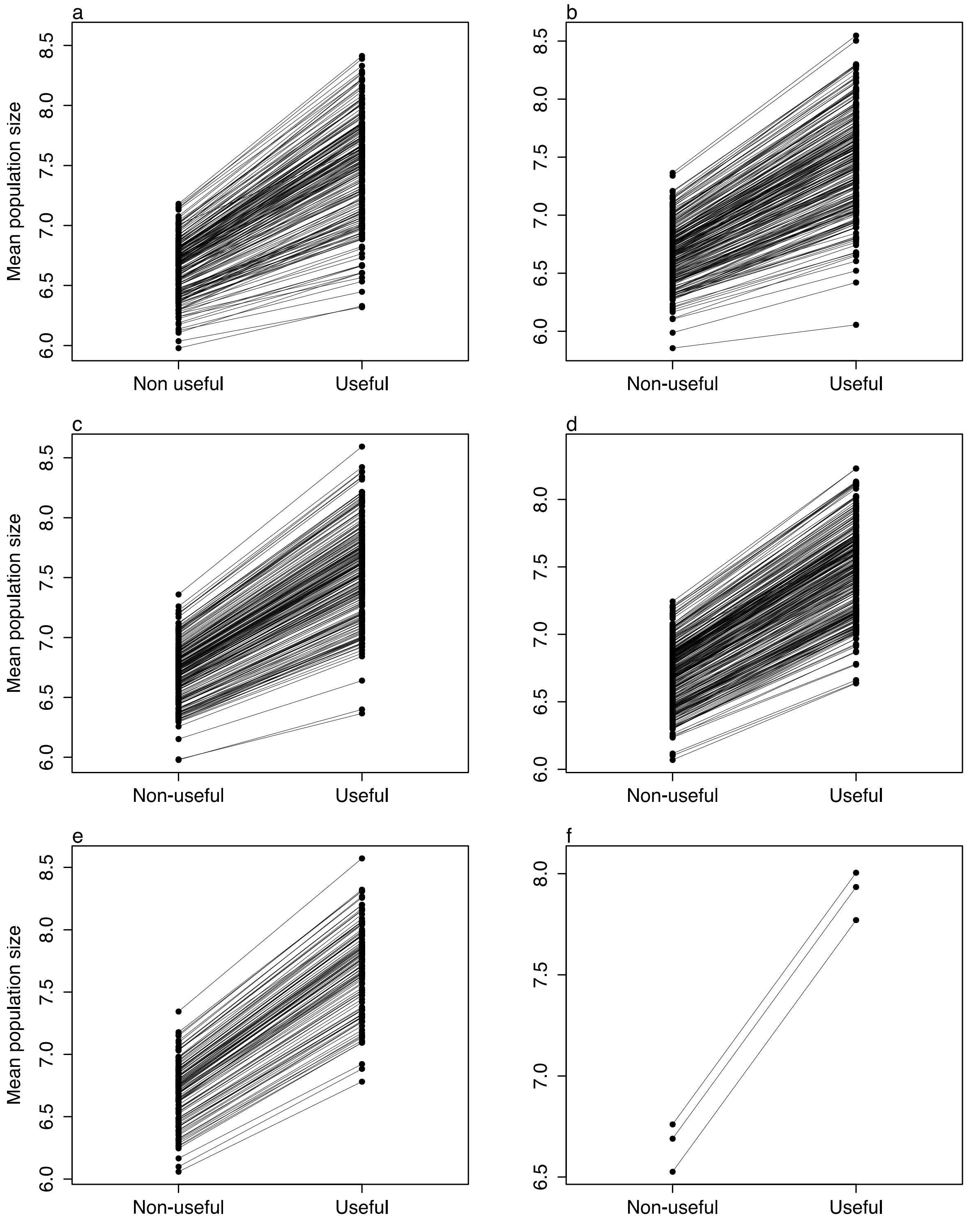

Supplement: S2 Fig — (PDF) [file pone.0257875.s004.pdf]
