# Supplementary material for: Eighty-four per cent of all Amazonian arboreal plant individuals are useful to humans
Source: PLoS One. 2021 Oct 1;16(10):e0257875. doi: 10.1371/journal.pone.0257875 (PMC8486103; doi:10.1371/journal.pone.0257875)

S3 Fig. Relationship between the mean population sizes of arboreal species and their use categories. Bootstraps show means and confidence intervals of population sizes of species based on their single use (green), multiple uses (black) and main use category (red). Single use: species is used in one use category and is reported in one use category. Multiple uses: species is used in more than one use category and is reported in more than one use category. Main use category: species may be used in more than one use category but we assigned them a main use category; the species is reported in only one use category. The bars represent 95 % confidence intervals.

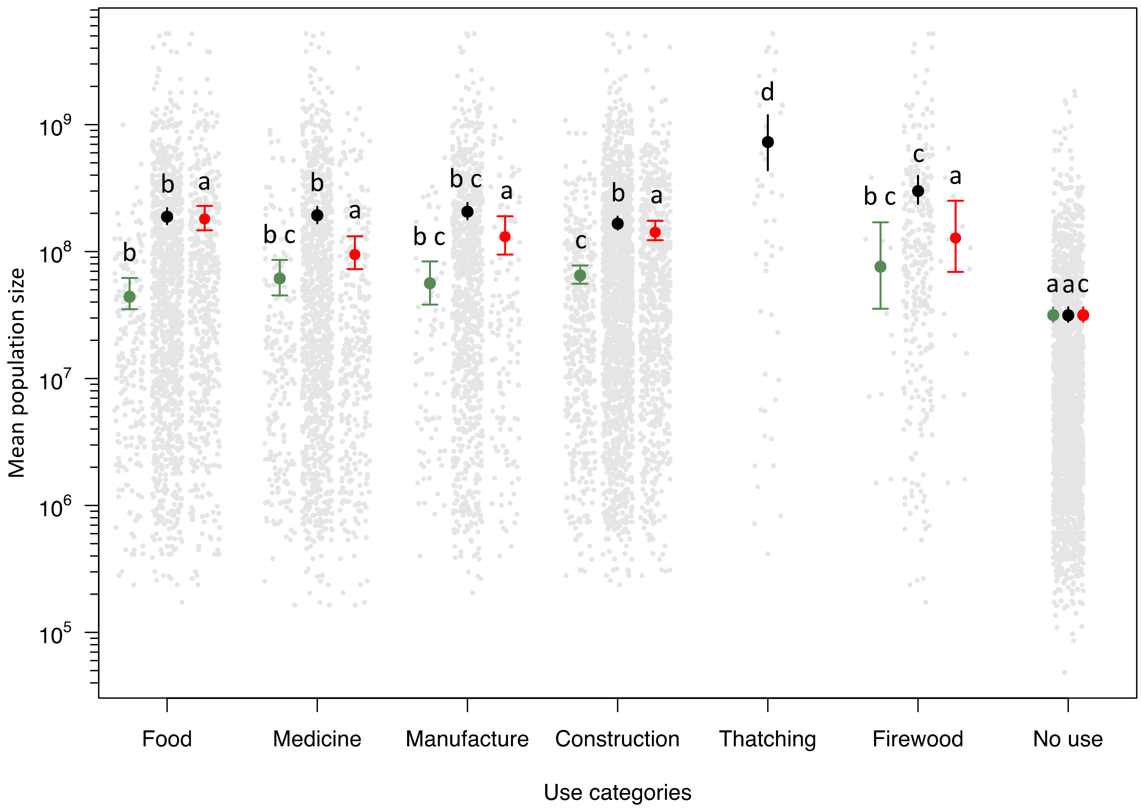

Supplement: S3 Fig — (PDF) [file pone.0257875.s005.pdf]
