# Supplementary material for: Eighty-four per cent of all Amazonian arboreal plant individuals are useful to humans
Source: PLoS One. 2021 Oct 1;16(10):e0257875. doi: 10.1371/journal.pone.0257875 (PMC8486103; doi:10.1371/journal.pone.0257875)

S4 Fig. Relationship between the mean population sizes of arboreal species and their number of uses, based on the number of use categories the species have (food, medicine, manufacturing, thatching, firewood and construction). Bootstraps show means and confidence intervals of population sizes of species. The bars represent 95 % confidence intervals.

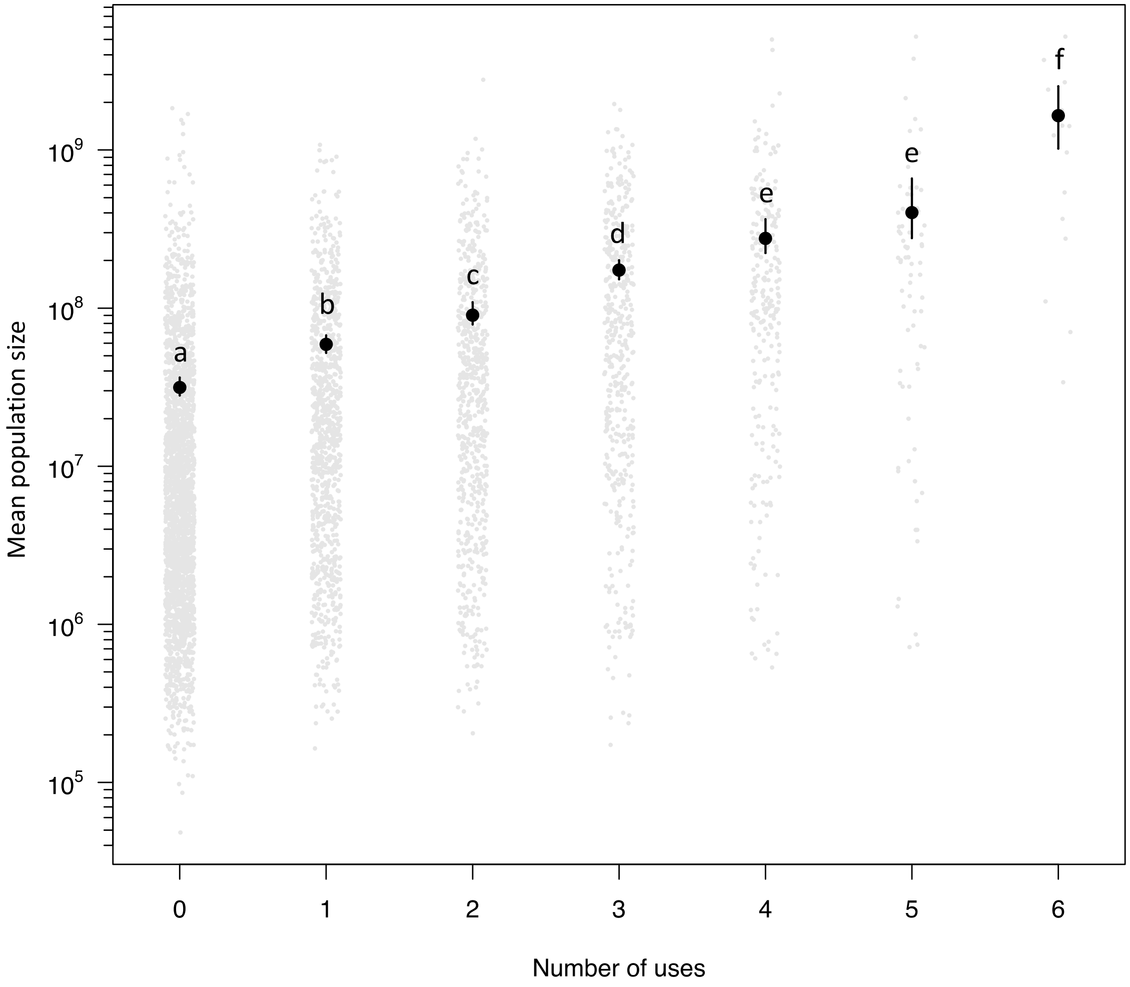

Supplement: S4 Fig — (PDF) [file pone.0257875.s006.pdf]
