# Supplementary material for: Eighty-four per cent of all Amazonian arboreal plant individuals are useful to humans
Source: PLoS One. 2021 Oct 1;16(10):e0257875. doi: 10.1371/journal.pone.0257875 (PMC8486103; doi:10.1371/journal.pone.0257875)
Supplement: S1 Table — (PDF) [file pone.0257875.s007.pdf]

S1 Table. Plant uses categories description, adapted from Prance et al. (1) and Macía et al. (2).

Use category	Description	Sub-categories	Description sub-categories
**Food**	Plants used for food consumption by humans	Food	Freshly, prepared and processed edible food. Includes stimulants to obtain energy for activities
		Food additives	Ingredients used in the preparation and processing of foods
		Beverages	Elaboration of unfermented or fermented drinks. Includes aromatic drink appreciated for its flavor
		Oils	Edible fats
**Medicine**	Plants used for physical and mental therapeutic purposes	Body health	Medicine to treat and prevent general ailments and human body diseases. Includes insect repellents
		Veterinary	Treatment of diseases or ailments for domestic animals
		Magic uses	Ailments or disorders of magic-religious origin recognized by a specific culture
		Ritual uses	Plants used for mental therapeutic purposes
**Construction**	Stem and trunks used for construction	Post-and-beam construction	Stems used to build houses, watercourses and other constructions such as temporary camps and animal yards. Wood staves for internal support of roof thatch. Stems used as posts, frames, posts, gutters to transport water. Split palm stems used for walls.
		Transportation	Stems used for naval constructions and transportation. Includes canoes, boards, rafts, oars, wood for ox-drawn cart bed
		Fences	Territorial delimitation
		Furniture	Furniture manufacturing
**Thatching**	Thatching made from leaves	Thatch	Thatching of houses and other constructions, such as improvised shelter
**Manufacturing**	Plants used for manufactures	Cosmetic and hygiene	Beauty products. Includes soap, perfum, oils and shampoo
		Dyes	Dyeing materials. Includes lashing material, dyes, glues, craft fibers, pottery temper, ink for body painting, craft ink
		Personal Adornment	Articles of clothing and accessories. Includes necklaces, bracelets, earrings, armbands, pectorals, anklets and hats
		Musical instruments	Materials or plant part used as musical instruments
		Toys	Materials or plant part used as toys
		Tools and weapons	Fishing and hunting tools, labour tools. Includes bows, arrows, paddles, harpoons, fishing nets, hunting traps, craft fibers, ropes, moorings, blowpipes
		Household items	Household equipment. Includes sifters, baskets, fans, hammocks, bags and air freshener
		Poisons	Poison for fishing, hunting and agriculture. Includes “curare”, pesticide and fertilizer
		Caulking and smoking	Materials used for caulking and smoking. Includes caulk canoe, rubber, glues, pottery temper and paper

**Firewood**	Stem and trunks used for firewood or charcoal	Firewood or charcoal	Stem and trunks used for firewood or charcoal

References

1. Prance GT, Balée W, Boom BM, Carneiro RL. Quantitative ethnobotany and the case for conservation in Amazonia. Conserv Biol. 1987;1(4):296–310.
2. Macía MJ, Armesilla PJ, Cámara-Leret R, Paniagua-Zambrana N, Villalba S, Balslev H, et al. Palm uses in northwestern South America: a quantitative review. Bot Rev. 2011;77(4):462–570.
